# Supplementary material for: Integration of Epidemiological and Genomic Data to Investigate H5N1 HPAI Outbreaks in Northern Italy in 2021–2022
Source: Pathogens. 2023 Jan 6;12(1):100. doi: 10.3390/pathogens12010100 (PMC9865711; doi:10.3390/pathogens12010100)
Supplement: Supplementary file 1 [file pathogens-12-00100-s001.zip › Supplementary Figure S1.pdf]

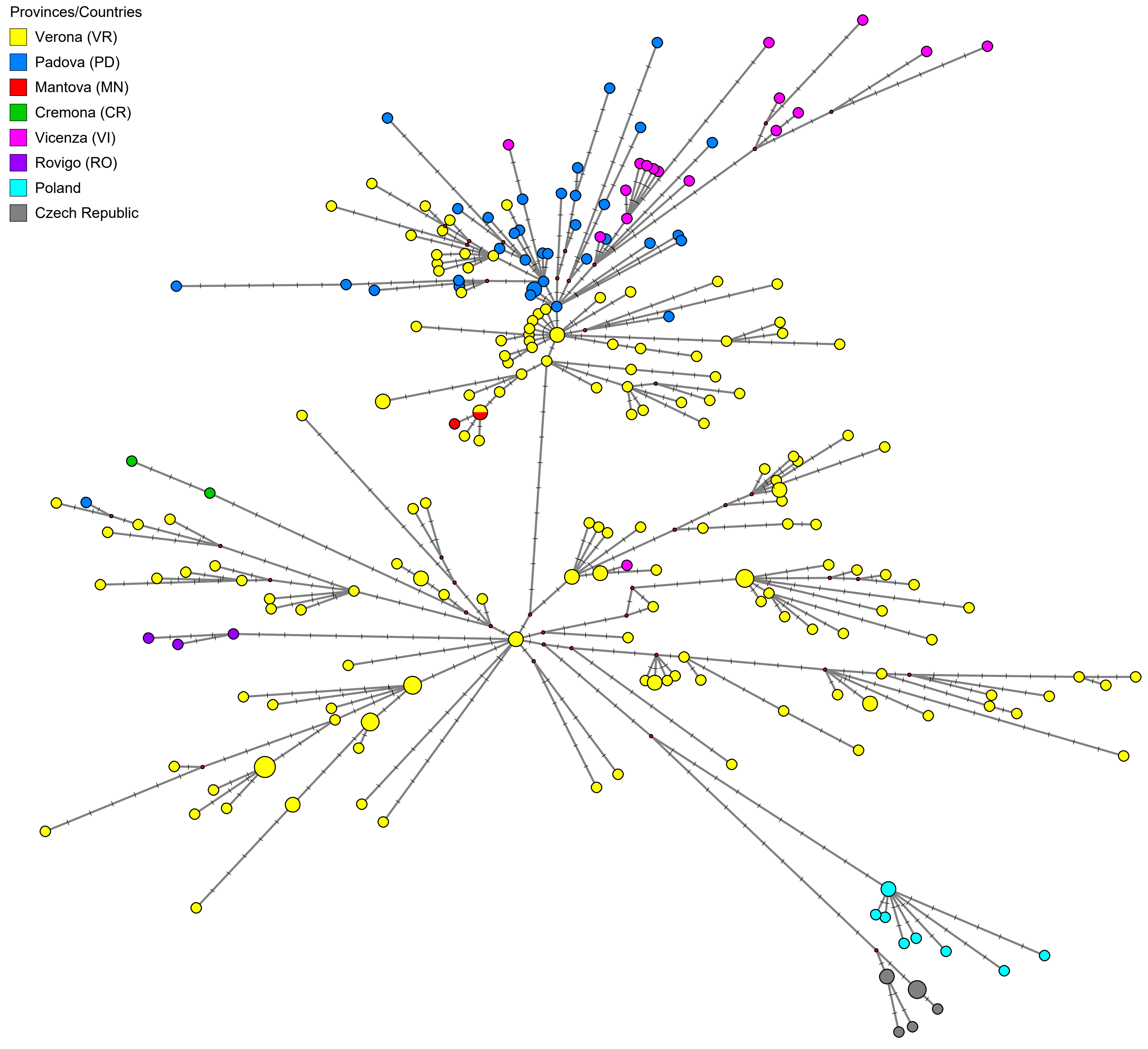
Provinces/Countries
Verona (VR)
Padova (PD)
Mantova (MN)
Cremona (CR)
Vicenza (VI)
Rovigo (RO)
Poland
Czech Republic
