# Supplementary material for: Integration of Epidemiological and Genomic Data to Investigate H5N1 HPAI Outbreaks in Northern Italy in 2021–2022
Source: Pathogens. 2023 Jan 6;12(1):100. doi: 10.3390/pathogens12010100 (PMC9865711; doi:10.3390/pathogens12010100)
Supplement: Supplementary file 1 [file pathogens-12-00100-s001.zip › Supplementary Table S1.pdf]

Supplementary Table S1. Accession numbers of the Italian viruses analyzed.

Isolate_Id	PB2	PB1	PA	HA	NP	NA	MP	NS	Isolate_Name	Present in the phylogenetic network	Subtype
EPI_ISL_14761428	EPI2141766	EPI2141768	EPI2141765	EPI2141770	EPI2141762	EPI2141769	EPI2141764	EPI2141763	A/goose/Italy/22VIR1520/2022		H5N1
EPI_ISL_14760656	EPI2140311	EPI2140312	EPI2140310	EPI2140314	EPI2140306	EPI2140313	EPI2140309	EPI2140308	A/goose/Italy/21VIR10458/2021	*	H5N1
EPI_ISL_14760611	EPI2139932	EPI2139933	EPI2139931	EPI2139935	EPI2139928	EPI2139934	EPI2139930	EPI2139929	A/goose/Italy/IZSLT_21VIR10273/2021		H5N1
EPI_ISL_7733645	EPI1944482	EPI1944483	EPI1944481	EPI1944485	EPI1944478	EPI1944484	EPI1944480	EPI1944479	A/goose/Italy/IZSLT-21VIR10273/2021		H5N1
EPI_ISL_14761291	EPI2141652	EPI2141653	EPI2141651	EPI2141655	EPI2141647	EPI2141654	EPI2141650	EPI2141649	A/guinea_fowl/Italy/22VIR205-4/2022		H5N1
EPI_ISL_14760762	EPI2141204	EPI2141205	EPI2141203	EPI2141208	EPI2141200	EPI2141206	EPI2141202	EPI2141201	A/guinea_fowl/Italy/21VIR11383-1/2021	*	H5N1
EPI_ISL_14760745	EPI2141083	EPI2141084	EPI2141082	EPI2141086	EPI2141078	EPI2141085	EPI2141080	EPI2141079	A/guinea_fowl/Italy/21VIR11298/2021	*	H5N1
EPI_ISL_14760638	EPI2140152	EPI2140153	EPI2140151	EPI2140155	EPI2140148	EPI2140154	EPI2140150	EPI2140149	A/guinea_fowl/Italy/21VIR10351/2021	*	H5N1
EPI_ISL_14760633	EPI2140112	EPI2140113	EPI2140111	EPI2140115	EPI2140108	EPI2140114	EPI2140110	EPI2140109	A/Guinea_fowl/Italy/21VIR10231/2021	*	H5N1
EPI_ISL_14760632	EPI2140104	EPI2140105	EPI2140103	EPI2140107	EPI2140100	EPI2140106	EPI2140102	EPI2140101	A/Guinea_fowl/Italy/21VIR10216/2021	*	H5N1
EPI_ISL_14761462	EPI2141793	EPI2141794	EPI2141792	EPI2141796	EPI2141788	EPI2141795	EPI2141791	EPI2141790	A/pheasant/Italy/22VIR1953-1/2022		H5N1
EPI_ISL_14760730	EPI2140952	EPI2140953	EPI2140951	EPI2140956	EPI2140948	EPI2140954	EPI2140950	EPI2140949	A/pheasant/Italy/21VIR11165/2021	*	H5N1
EPI_ISL_14760728	EPI2140935	EPI2140936	EPI2140934	EPI2140938	EPI2140930	EPI2140937	EPI2140933	EPI2140932	A/pheasant/Italy/21VIR11162/2021	*	H5N1
EPI_ISL_14760637	EPI2140144	EPI2140145	EPI2140143	EPI2140147	EPI2140140	EPI2140146	EPI2140142	EPI2140141	A/pheasant/Italy/21VIR10359/2021	*	H5N1
EPI_ISL_14761383	EPI2141731	EPI2141732	EPI2141730	EPI2141735	EPI2141727	EPI2141733	EPI2141729	EPI2141728	A/swan/Italy/22VIR1560/2022		H5N1
EPI_ISL_14761281	EPI2141643	EPI2141644	EPI2141642	EPI2141646	EPI2141639	EPI2141645	EPI2141641	EPI2141640	A/turkey/Italy/22VIR171-2/2022		H5N1
EPI_ISL_14761232	EPI2141581	EPI2141582	EPI2141580	EPI2141585	EPI2141577	EPI2141583	EPI2141579	EPI2141578	A/turkey/Italy/21VIR11935/2021		H5N1
EPI_ISL_14761199	EPI2141555	EPI2141556	EPI2141554	EPI2141558	EPI2141550	EPI2141557	EPI2141552	EPI2141551	A/turkey/Italy/21VIR11772-7/2021		H5N1
EPI_ISL_14761152	EPI2141519	EPI2141520	EPI2141518	EPI2141523	EPI2141515	EPI2141522	EPI2141517	EPI2141516	A/turkey/Italy/21VIR11661-7/2021	*	H5N1
EPI_ISL_14761141	EPI2141511	EPI2141512	EPI2141510	EPI2141514	EPI2141506	EPI2141513	EPI2141509	EPI2141507	A/turkey/Italy/21VIR11690/2021	*	H5N1
EPI_ISL_14761129	EPI2141502	EPI2141503	EPI2141501	EPI2141505	EPI2141498	EPI2141504	EPI2141500	EPI2141499	A/turkey/Italy/21VIR11692/2021	*	H5N1
EPI_ISL_14761119	EPI2141493	EPI2141494	EPI2141492	EPI2141496	EPI2141489	EPI2141495	EPI2141491	EPI2141490	A/turkey/Italy/21VIR11691/2021	*	H5N1
EPI_ISL_14761107	EPI2141484	EPI2141485	EPI2141483	EPI2141488	EPI2141480	EPI2141487	EPI2141482	EPI2141481	A/turkey/Italy/21VIR11587-2/2021		H5N1
EPI_ISL_14761097	EPI2141475	EPI2141477	EPI2141474	EPI2141479	EPI2141471	EPI2141478	EPI2141473	EPI2141472	A/turkey/Italy/21VIR11587-1/2021	*	H5N1
EPI_ISL_14761086	EPI2141467	EPI2141468	EPI2141466	EPI2141470	EPI2141462	EPI2141469	EPI2141464	EPI2141463	A/turkey/Italy/21VIR11586-1/2021	*	H5N1
EPI_ISL_14761031	EPI2141423	EPI2141424	EPI2141422	EPI2141426	EPI2141418	EPI2141425	EPI2141420	EPI2141419	A/turkey/Italy/21VIR11596-3/2021		H5N1
EPI_ISL_14760999	EPI2141396	EPI2141397	EPI2141395	EPI2141400	EPI2141392	EPI2141398	EPI2141394	EPI2141393	A/turkey/Italy/21VIR11504/2021		H5N1
EPI_ISL_14760977	EPI2141379	EPI2141380	EPI2141377	EPI2141382	EPI2141374	EPI2141381	EPI2141376	EPI2141375	A/turkey/Italy/21VIR11500-3/2021		H5N1
EPI_ISL_14760967	EPI2141370	EPI2141371	EPI2141369	EPI2141373	EPI2141365	EPI2141372	EPI2141368	EPI2141367	A/turkey/Italy/21VIR11495-5/2021	*	H5N1
EPI_ISL_14760956	EPI2141361	EPI2141362	EPI2141360	EPI2141364	EPI2141357	EPI2141363	EPI2141359	EPI2141358	A/turkey/Italy/21VIR11492-4/2021	*	H5N1
EPI_ISL_14760923	EPI2141335	EPI2141336	EPI2141334	EPI2141338	EPI2141330	EPI2141337	EPI2141332	EPI2141331	A/turkey/Italy/21VIR11503-1/2021		H5N1
EPI_ISL_14760913	EPI2141326	EPI2141327	EPI2141325	EPI2141329	EPI2141322	EPI2141328	EPI2141324	EPI2141323	A/turkey/Italy/21VIR11499-5/2021		H5N1
EPI_ISL_14760902	EPI2141317	EPI2141318	EPI2141316	EPI2141321	EPI2141313	EPI2141319	EPI2141315	EPI2141314	A/turkey/Italy/21VIR11506/2021	*	H5N1
EPI_ISL_14760891	EPI2141308	EPI2141310	EPI2141307	EPI2141312	EPI2141304	EPI2141311	EPI2141306	EPI2141305	A/turkey/Italy/21VIR11511/2021	*	H5N1
EPI_ISL_14760870	EPI2141291	EPI2141292	EPI2141290	EPI2141295	EPI2141287	EPI2141293	EPI2141289	EPI2141288	A/turkey/Italy/21VIR11440-2/2021	*	H5N1
EPI_ISL_14760859	EPI2141282	EPI2141283	EPI2141281	EPI2141286	EPI2141278	EPI2141285	EPI2141280	EPI2141279	A/turkey/Italy/21VIR11510/2021		H5N1

Isolate_Id	PB2	PB1	PA	HA	NP	NA	MP	NS	Isolate_Name	Present in the phylogenetic network	Subtype
EPI_ISL_14760849	EPI2141274	EPI2141275	EPI2141273	EPI2141277	EPI2141270	EPI2141276	EPI2141272	EPI2141271	A/turkey/Italy/21VIR11509/2021		H5N1
EPI_ISL_14760814	EPI2141248	EPI2141249	EPI2141247	EPI2141251	EPI2141243	EPI2141250	EPI2141246	EPI2141244	A/turkey/Italy/21VIR11396-1/2021		H5N1
EPI_ISL_14760784	EPI2141222	EPI2141223	EPI2141220	EPI2141225	EPI2141217	EPI2141224	EPI2141219	EPI2141218	A/turkey/Italy/21VIR11386-1/2021	*	H5N1
EPI_ISL_14760758	EPI2141195	EPI2141197	EPI2141194	EPI2141199	EPI2141191	EPI2141198	EPI2141193	EPI2141192	A/turkey/Italy/21VIR11393-3/2021		H5N1
EPI_ISL_14760757	EPI2141187	EPI2141188	EPI2141186	EPI2141190	EPI2141183	EPI2141189	EPI2141185	EPI2141184	A/turkey/Italy/21VIR11392-2/2021		H5N1
EPI_ISL_14760756	EPI2141178	EPI2141179	EPI2141177	EPI2141181	EPI2141174	EPI2141180	EPI2141176	EPI2141175	A/turkey/Italy/21VIR11391-3/2021		H5N1
EPI_ISL_14760754	EPI2141161	EPI2141162	EPI2141160	EPI2141164	EPI2141157	EPI2141163	EPI2141159	EPI2141158	A/turkey/Italy/21VIR11387-1/2021		H5N1
EPI_ISL_14760753	EPI2141152	EPI2141153	EPI2141151	EPI2141155	EPI2141148	EPI2141154	EPI2141150	EPI2141149	A/turkey/Italy/21VIR11384-1/2021		H5N1
EPI_ISL_14760749	EPI2141117	EPI2141118	EPI2141116	EPI2141121	EPI2141113	EPI2141119	EPI2141115	EPI2141114	A/turkey/Italy/21VIR11254-1/2021		H5N1
EPI_ISL_14760748	EPI2141109	EPI2141110	EPI2141108	EPI2141112	EPI2141104	EPI2141111	EPI2141107	EPI2141106	A/turkey/Italy/21VIR11252-1/2021		H5N1
EPI_ISL_14760747	EPI2141100	EPI2141101	EPI2141099	EPI2141103	EPI2141096	EPI2141102	EPI2141098	EPI2141097	A/turkey/Italy/21VIR11251-1/2021		H5N1
EPI_ISL_14760746	EPI2141091	EPI2141092	EPI2141090	EPI2141095	EPI2141087	EPI2141094	EPI2141089	EPI2141088	A/turkey/Italy/21VIR11302/2021	*	H5N1
EPI_ISL_14760743	EPI2141065	EPI2141066	EPI2141064	EPI2141069	EPI2141061	EPI2141067	EPI2141063	EPI2141062	A/turkey/Italy/21VIR11237/2021	*	H5N1
EPI_ISL_14760740	EPI2141039	EPI2141040	EPI2141038	EPI2141043	EPI2141035	EPI2141041	EPI2141037	EPI2141036	A/turkey/Italy/21VIR11163/2021	*	H5N1
EPI_ISL_14760739	EPI2141030	EPI2141032	EPI2141029	EPI2141034	EPI2141026	EPI2141033	EPI2141028	EPI2141027	A/turkey/Italy/21VIR11058/2021	*	H5N1
EPI_ISL_14760729	EPI2140943	EPI2140944	EPI2140942	EPI2140947	EPI2140939	EPI2140946	EPI2140941	EPI2140940	A/turkey/Italy/21VIR11164/2021	*	H5N1
EPI_ISL_14760726	EPI2140917	EPI2140918	EPI2140916	EPI2140921	EPI2140913	EPI2140920	EPI2140915	EPI2140914	A/turkey/Italy/21VIR11166/2021		H5N1
EPI_ISL_14760725	EPI2140909	EPI2140910	EPI2140908	EPI2140912	EPI2140904	EPI2140911	EPI2140906	EPI2140905	A/turkey/Italy/21VIR11007-1/2021	*	H5N1
EPI_ISL_14760724	EPI2140900	EPI2140901	EPI2140899	EPI2140903	EPI2140896	EPI2140902	EPI2140898	EPI2140897	A/turkey/Italy/21VIR11006-1/2021	*	H5N1
EPI_ISL_14760720	EPI2140865	EPI2140866	EPI2140864	EPI2140869	EPI2140861	EPI2140867	EPI2140863	EPI2140862	A/turkey/Italy/21VIR11077-1/2021	*	H5N1
EPI_ISL_14760708	EPI2140761	EPI2140762	EPI2140760	EPI2140764	EPI2140756	EPI2140763	EPI2140759	EPI2140758	A/turkey/Italy/21VIR10988-1/2021	*	H5N1
EPI_ISL_14760703	EPI2140717	EPI2140718	EPI2140716	EPI2140721	EPI2140713	EPI2140719	EPI2140715	EPI2140714	A/turkey/Italy/21VIR10970-3/2021		H5N1
EPI_ISL_14760702	EPI2140709	EPI2140710	EPI2140708	EPI2140712	EPI2140704	EPI2140711	EPI2140706	EPI2140705	A/turkey/Italy/21VIR10847-1/2021	*	H5N1
EPI_ISL_14760700	EPI2140691	EPI2140692	EPI2140690	EPI2140695	EPI2140687	EPI2140693	EPI2140689	EPI2140688	A/turkey/Italy/21VIR10757-1/2021	*	H5N1
EPI_ISL_14760699	EPI2140683	EPI2140684	EPI2140681	EPI2140686	EPI2140678	EPI2140685	EPI2140680	EPI2140679	A/turkey/Italy/21VIR10930/2021	*	H5N1
EPI_ISL_14760697	EPI2140665	EPI2140666	EPI2140664	EPI2140669	EPI2140661	EPI2140667	EPI2140663	EPI2140662	A/turkey/Italy/21VIR10929/2021	*	H5N1
EPI_ISL_14760694	EPI2140638	EPI2140640	EPI2140637	EPI2140642	EPI2140634	EPI2140641	EPI2140636	EPI2140635	A/turkey/Italy/21VIR10810-1/2021		H5N1
EPI_ISL_14760690	EPI2140603	EPI2140604	EPI2140602	EPI2140607	EPI2140599	EPI2140605	EPI2140601	EPI2140600	A/turkey/Italy/21VIR10737-1/2021	*	H5N1
EPI_ISL_14760689	EPI2140594	EPI2140595	EPI2140593	EPI2140598	EPI2140590	EPI2140596	EPI2140592	EPI2140591	A/turkey/Italy/21VIR10697-1/2021	*	H5N1
EPI_ISL_14760687	EPI2140576	EPI2140578	EPI2140575	EPI2140580	EPI2140572	EPI2140579	EPI2140574	EPI2140573	A/turkey/Italy/21VIR10855/2021	*	H5N1
EPI_ISL_14760686	EPI2140568	EPI2140569	EPI2140567	EPI2140571	EPI2140563	EPI2140570	EPI2140565	EPI2140564	A/turkey/Italy/21VIR10854/2021	*	H5N1
EPI_ISL_14760685	EPI2140559	EPI2140560	EPI2140558	EPI2140562	EPI2140555	EPI2140561	EPI2140557	EPI2140556	A/turkey/Italy/21VIR10857/2021	*	H5N1
EPI_ISL_14760684	EPI2140550	EPI2140551	EPI2140549	EPI2140553	EPI2140546	EPI2140552	EPI2140548	EPI2140547	A/turkey/Italy/21VIR10852/2021	*	H5N1
EPI_ISL_14760683	EPI2140541	EPI2140542	EPI2140540	EPI2140545	EPI2140537	EPI2140543	EPI2140539	EPI2140538	A/turkey/Italy/21VIR10856/2021	*	H5N1
EPI_ISL_14760682	EPI2140532	EPI2140533	EPI2140531	EPI2140536	EPI2140528	EPI2140534	EPI2140530	EPI2140529	A/turkey/Italy/21VIR10699-1/2021	*	H5N1
EPI_ISL_14760680	EPI2140514	EPI2140516	EPI2140513	EPI2140518	EPI2140510	EPI2140517	EPI2140512	EPI2140511	A/turkey/Italy/21VIR10645-15/2021	*	H5N1
EPI_ISL_14760679	EPI2140506	EPI2140507	EPI2140505	EPI2140509	EPI2140501	EPI2140508	EPI2140504	EPI2140502	A/turkey/Italy/21VIR10636-17/2021	*	H5N1

Isolate_Id	PB2	PB1	PA	HA	NP	NA	MP	NS	Isolate_Name	Present in the phylogenetic network	Subtype
EPI_ISL_14760669	EPI2140417	EPI2140418	EPI2140416	EPI2140421	EPI2140413	EPI2140419	EPI2140415	EPI2140414	A/turkey/Italy/21VIR10621-1/2021	*	H5N1
EPI_ISL_14760668	EPI2140408	EPI2140409	EPI2140407	EPI2140412	EPI2140404	EPI2140410	EPI2140406	EPI2140405	A/turkey/Italy/21VIR10617-2/2021	*	H5N1
EPI_ISL_14760667	EPI2140399	EPI2140400	EPI2140398	EPI2140403	EPI2140395	EPI2140402	EPI2140397	EPI2140396	A/turkey/Italy/21VIR10622-1/2021	*	H5N1
EPI_ISL_14760664	EPI2140373	EPI2140374	EPI2140372	EPI2140376	EPI2140368	EPI2140375	EPI2140371	EPI2140370	A/turkey/Italy/21VIR10459-1/2021	*	H5N1
EPI_ISL_14760663	EPI2140364	EPI2140365	EPI2140363	EPI2140367	EPI2140360	EPI2140366	EPI2140362	EPI2140361	A/turkey/Italy/21VIR10524-1/2021		H5N1
EPI_ISL_14760660	EPI2140337	EPI2140338	EPI2140336	EPI2140341	EPI2140333	EPI2140340	EPI2140335	EPI2140334	A/turkey/Italy/21VIR10471/2021	*	H5N1
EPI_ISL_14760653	EPI2140284	EPI2140285	EPI2140283	EPI2140288	EPI2140280	EPI2140286	EPI2140282	EPI2140281	A/turkey/Italy/21VIR10398/2021	*	H5N1
EPI_ISL_14760652	EPI2140275	EPI2140276	EPI2140274	EPI2140279	EPI2140271	EPI2140278	EPI2140273	EPI2140272	A/turkey/Italy/21VIR10397/2021	*	H5N1
EPI_ISL_14760650	EPI2140258	EPI2140259	EPI2140256	EPI2140261	EPI2140253	EPI2140260	EPI2140255	EPI2140254	A/turkey/Italy/21VIR10395/2021	*	H5N1
EPI_ISL_14760649	EPI2140249	EPI2140250	EPI2140248	EPI2140252	EPI2140244	EPI2140251	EPI2140247	EPI2140245	A/turkey/Italy/21VIR10396/2021	*	H5N1
EPI_ISL_14760647	EPI2140231	EPI2140232	EPI2140230	EPI2140234	EPI2140227	EPI2140233	EPI2140229	EPI2140228	A/turkey/Italy/21VIR10338/2021	*	H5N1
EPI_ISL_14760634	EPI2140120	EPI2140121	EPI2140119	EPI2140123	EPI2140116	EPI2140122	EPI2140118	EPI2140117	A/turkey/Italy/21VIR10103/2021	*	H5N1
EPI_ISL_14760631	EPI2140096	EPI2140097	EPI2140095	EPI2140099	EPI2140092	EPI2140098	EPI2140094	EPI2140093	A/turkey/Italy/21VIR9901-3/2021	*	H5N1
EPI_ISL_14760630	EPI2140088	EPI2140089	EPI2140087	EPI2140091	EPI2140084	EPI2140090	EPI2140086	EPI2140085	A/turkey/Italy/21VIR10092/2021	*	H5N1
EPI_ISL_14760628	EPI2140072	EPI2140073	EPI2140071	EPI2140075	EPI2140067	EPI2140074	EPI2140070	EPI2140069	A/turkey/Italy/21VIR10095/2021	*	H5N1
EPI_ISL_14760627	EPI2140062	EPI2140063	EPI2140061	EPI2140066	EPI2140058	EPI2140064	EPI2140060	EPI2140059	A/turkey/Italy/21VIR10097/2021	*	H5N1
EPI_ISL_14760623	EPI2140028	EPI2140029	EPI2140027	EPI2140031	EPI2140024	EPI2140030	EPI2140026	EPI2140025	A/turkey/Italy/21VIR9949-9/2021	*	H5N1
EPI_ISL_14760621	EPI2140012	EPI2140013	EPI2140011	EPI2140015	EPI2140008	EPI2140014	EPI2140010	EPI2140009	A/turkey/Italy/21VIR9894-3/2021	*	H5N1
EPI_ISL_14760619	EPI2139996	EPI2139997	EPI2139995	EPI2139999	EPI2139992	EPI2139998	EPI2139994	EPI2139993	A/turkey/Italy/21VIR10001/2021	*	H5N1
EPI_ISL_14760618	EPI2139988	EPI2139989	EPI2139987	EPI2139991	EPI2139984	EPI2139990	EPI2139986	EPI2139985	A/turkey/Italy/21VIR9997/2021	*	H5N1
EPI_ISL_14760617	EPI2139980	EPI2139981	EPI2139979	EPI2139983	EPI2139976	EPI2139982	EPI2139978	EPI2139977	A/turkey/Italy/21VIR9999/2021	*	H5N1
EPI_ISL_14760615	EPI2139964	EPI2139965	EPI2139963	EPI2139967	EPI2139960	EPI2139966	EPI2139962	EPI2139961	A/turkey/Italy/21VIR9996/2021	*	H5N1
EPI_ISL_14760614	EPI2139956	EPI2139957	EPI2139955	EPI2139959	EPI2139952	EPI2139958	EPI2139954	EPI2139953	A/turkey/Italy/21VIR9891-21/2021	*	H5N1
EPI_ISL_14760613	EPI2139948	EPI2139949	EPI2139947	EPI2139951	EPI2139944	EPI2139950	EPI2139946	EPI2139945	A/turkey/Italy/21VIR9889-1/2021	*	H5N1
EPI_ISL_14760609	EPI2139917	EPI2139918	EPI2139916	EPI2139920	EPI2139913	EPI2139919	EPI2139915	EPI2139914	A/turkey/Italy/21VIR9839-1/2021	*	H5N1
EPI_ISL_14760607	EPI2139901	EPI2139902	EPI2139900	EPI2139904	EPI2139897	EPI2139903	EPI2139899	EPI2139898	A/turkey/Italy/21VIR9905-2/2021	*	H5N1
EPI_ISL_14760606	EPI2139893	EPI2139894	EPI2139892	EPI2139896	EPI2139889	EPI2139895	EPI2139891	EPI2139890	A/turkey/Italy/21VIR9906-2/2021	*	H5N1
EPI_ISL_14760601	EPI2139853	EPI2139854	EPI2139852	EPI2139856	EPI2139849	EPI2139855	EPI2139851	EPI2139850	A/turkey/Italy/21VIR9903-2/2021	*	H5N1
EPI_ISL_14760600	EPI2139845	EPI2139846	EPI2139844	EPI2139848	EPI2139841	EPI2139847	EPI2139843	EPI2139842	A/turkey/Italy/21VIR9869-4/2021	*	H5N1
EPI_ISL_14760599	EPI2139837	EPI2139838	EPI2139836	EPI2139840	EPI2139833	EPI2139839	EPI2139835	EPI2139834	A/turkey/Italy/21VIR9870-3/2021	*	H5N1
EPI_ISL_14760596	EPI2139813	EPI2139814	EPI2139812	EPI2139816	EPI2139809	EPI2139815	EPI2139811	EPI2139810	A/turkey/Italy/21VIR9766-3/2021	*	H5N1
EPI_ISL_14760595	EPI2139805	EPI2139806	EPI2139804	EPI2139808	EPI2139801	EPI2139807	EPI2139803	EPI2139802	A/turkey/Italy/21VIR9769-4/2021	*	H5N1
EPI_ISL_14760594	EPI2139797	EPI2139798	EPI2139796	EPI2139800	EPI2139793	EPI2139799	EPI2139795	EPI2139794	A/turkey/Italy/21VIR9697-3/2021	*	H5N1
EPI_ISL_14760593	EPI2139789	EPI2139790	EPI2139788	EPI2139792	EPI2139785	EPI2139791	EPI2139787	EPI2139786	A/turkey/Italy/21VIR9696-2/2021	*	H5N1
EPI_ISL_14760592	EPI2139781	EPI2139782	EPI2139780	EPI2139784	EPI2139777	EPI2139783	EPI2139779	EPI2139778	A/turkey/Italy/21VIR9693-3/2021	*	H5N1
EPI_ISL_14760591	EPI2139773	EPI2139774	EPI2139772	EPI2139776	EPI2139769	EPI2139775	EPI2139771	EPI2139770	A/turkey/Italy/21VIR9695-4/2021	*	H5N1
EPI_ISL_14760590	EPI2139765	EPI2139766	EPI2139764	EPI2139768	EPI2139761	EPI2139767	EPI2139763	EPI2139762	A/turkey/Italy/21VIR9692-16/2021	*	H5N1

Isolate_Id	PB2	PB1	PA	HA	NP	NA	MP	NS	Isolate_Name	Present in the phylogenetic network	Subtype
EPI_ISL_14760589	EPI2139757	EPI2139758	EPI2139756	EPI2139760	EPI2139753	EPI2139759	EPI2139755	EPI2139754	A/turkey/Italy/21VIR9694-2/2021	*	H5N1
EPI_ISL_14760588	EPI2139749	EPI2139750	EPI2139748	EPI2139752	EPI2139745	EPI2139751	EPI2139747	EPI2139746	A/turkey/Italy/21VIR9647-2/2021	*	H5N1
EPI_ISL_14760587	EPI2139741	EPI2139742	EPI2139740	EPI2139744	EPI2139737	EPI2139743	EPI2139739	EPI2139738	A/turkey/Italy/21VIR9646-2/2021	*	H5N1
EPI_ISL_14760586	EPI2139733	EPI2139734	EPI2139732	EPI2139736	EPI2139729	EPI2139735	EPI2139731	EPI2139730	A/turkey/Italy/21VIR9650-2/2021	*	H5N1
EPI_ISL_14760585	EPI2139725	EPI2139726	EPI2139724	EPI2139728	EPI2139721	EPI2139727	EPI2139723	EPI2139722	A/turkey/Italy/21VIR9651-2/2021	*	H5N1
EPI_ISL_14760584	EPI2139717	EPI2139718	EPI2139716	EPI2139720	EPI2139713	EPI2139719	EPI2139715	EPI2139714	A/turkey/Italy/21VIR9648-2/2021	*	H5N1
EPI_ISL_14760583	EPI2139709	EPI2139710	EPI2139708	EPI2139712	EPI2139705	EPI2139711	EPI2139707	EPI2139706	A/turkey/Italy/21VIR9579-10/2021	*	H5N1
EPI_ISL_14760581	EPI2139693	EPI2139694	EPI2139692	EPI2139696	EPI2139689	EPI2139695	EPI2139691	EPI2139690	A/turkey/Italy/21VIR9645-2/2021	*	H5N1
EPI_ISL_14760580	EPI2139685	EPI2139686	EPI2139684	EPI2139688	EPI2139681	EPI2139687	EPI2139683	EPI2139682	A/turkey/Italy/21VIR9644-2/2021	*	H5N1
EPI_ISL_14760579	EPI2139677	EPI2139678	EPI2139676	EPI2139680	EPI2139673	EPI2139679	EPI2139675	EPI2139674	A/turkey/Italy/21VIR9643-2/2021	*	H5N1
EPI_ISL_14760578	EPI2139669	EPI2139670	EPI2139668	EPI2139672	EPI2139665	EPI2139671	EPI2139667	EPI2139666	A/turkey/Italy/21VIR9520-1/2021	*	H5N1
EPI_ISL_14760577	EPI2139661	EPI2139662	EPI2139660	EPI2139664	EPI2139657	EPI2139663	EPI2139659	EPI2139658	A/turkey/Italy/21VIR9519-1/2021	*	H5N1
EPI_ISL_14760576	EPI2139653	EPI2139654	EPI2139652	EPI2139656	EPI2139649	EPI2139655	EPI2139651	EPI2139650	A/turkey/Italy/21VIR9518-1/2021	*	H5N1
EPI_ISL_14760574	EPI2139637	EPI2139638	EPI2139636	EPI2139640	EPI2139633	EPI2139639	EPI2139635	EPI2139634	A/turkey/Italy/21VIR9608-2/2021	*	H5N1
EPI_ISL_14760573	EPI2139629	EPI2139630	EPI2139628	EPI2139632	EPI2139625	EPI2139631	EPI2139627	EPI2139626	A/turkey/Italy/21VIR9605-4/2021	*	H5N1
EPI_ISL_14760572	EPI2139621	EPI2139622	EPI2139620	EPI2139624	EPI2139617	EPI2139623	EPI2139619	EPI2139618	A/turkey/Italy/21VIR9609-2/2021	*	H5N1
EPI_ISL_14760571	EPI2139613	EPI2139614	EPI2139612	EPI2139616	EPI2139609	EPI2139615	EPI2139611	EPI2139610	A/turkey/Italy/21VIR9604-3/2021	*	H5N1
EPI_ISL_14760570	EPI2139605	EPI2139606	EPI2139604	EPI2139608	EPI2139601	EPI2139607	EPI2139603	EPI2139602	A/turkey/Italy/21VIR9606-2/2021	*	H5N1
EPI_ISL_14760569	EPI2139597	EPI2139598	EPI2139596	EPI2139600	EPI2139593	EPI2139599	EPI2139595	EPI2139594	A/turkey/Italy/21VIR9607-2/2021	*	H5N1
EPI_ISL_14760565	EPI2139565	EPI2139566	EPI2139564	EPI2139568	EPI2139561	EPI2139567	EPI2139563	EPI2139562	A/turkey/Italy/21VIR9475-1/2021	*	H5N1
EPI_ISL_14760564	EPI2139557	EPI2139558	EPI2139556	EPI2139560	EPI2139553	EPI2139559	EPI2139555	EPI2139554	A/turkey/Italy/21VIR9476-1/2021	*	H5N1
EPI_ISL_14760563	EPI2139549	EPI2139550	EPI2139548	EPI2139552	EPI2139545	EPI2139551	EPI2139547	EPI2139546	A/turkey/Italy/21VIR9426-3/2021	*	H5N1
EPI_ISL_14760560	EPI2139525	EPI2139526	EPI2139524	EPI2139528	EPI2139521	EPI2139527	EPI2139523	EPI2139522	A/turkey/Italy/21VIR9372-1/2021	*	H5N1
EPI_ISL_14760556	EPI2139493	EPI2139494	EPI2139492	EPI2139496	EPI2139489	EPI2139495	EPI2139491	EPI2139490	A/turkey/Italy/21VIR9215-1/2021	*	H5N1
EPI_ISL_14760555	EPI2139485	EPI2139486	EPI2139484	EPI2139488	EPI2139481	EPI2139487	EPI2139483	EPI2139482	A/turkey/Italy/21VIR9213-1/2021	*	H5N1
EPI_ISL_14760554	EPI2139477	EPI2139478	EPI2139476	EPI2139480	EPI2139473	EPI2139479	EPI2139475	EPI2139474	A/turkey/Italy/21VIR9217-1/2021	*	H5N1
EPI_ISL_14760553	EPI2139469	EPI2139470	EPI2139468	EPI2139472	EPI2139465	EPI2139471	EPI2139467	EPI2139466	A/turkey/Italy/21VIR9211-1/2021	*	H5N1
EPI_ISL_14760551	EPI2139453	EPI2139454	EPI2139452	EPI2139456	EPI2139449	EPI2139455	EPI2139451	EPI2139450	A/turkey/Italy/21VIR9209-1/2021	*	H5N1
EPI_ISL_14760549	EPI2139437	EPI2139438	EPI2139436	EPI2139440	EPI2139433	EPI2139439	EPI2139435	EPI2139434	A/turkey/Italy/21VIR9144-2/2021	*	H5N1
EPI_ISL_14760547	EPI2139421	EPI2139422	EPI2139420	EPI2139424	EPI2139417	EPI2139423	EPI2139419	EPI2139418	A/turkey/Italy/21VIR9073-3/2021	*	H5N1
EPI_ISL_14760546	EPI2139413	EPI2139414	EPI2139412	EPI2139416	EPI2139409	EPI2139415	EPI2139411	EPI2139410	A/turkey/Italy/21VIR8926-2/2021		H5N1
EPI_ISL_14760545	EPI2139405	EPI2139406	EPI2139404	EPI2139408	EPI2139401	EPI2139407	EPI2139403	EPI2139402	A/turkey/Italy/21VIR8926-1/2021	*	H5N1
EPI_ISL_14760544	EPI2139397	EPI2139398	EPI2139396	EPI2139400	EPI2139393	EPI2139399	EPI2139395	EPI2139394	A/turkey/Italy/21VIR8925-6/2021	*	H5N1
EPI_ISL_14760543	EPI2139389	EPI2139390	EPI2139388	EPI2139392	EPI2139385	EPI2139391	EPI2139387	EPI2139386	A/turkey/Italy/21VIR8816-1/2021	*	H5N1
EPI_ISL_14760542	EPI2139381	EPI2139382	EPI2139380	EPI2139384	EPI2139377	EPI2139383	EPI2139379	EPI2139378	A/turkey/Italy/21VIR8728-1/2021	*	H5N1
EPI_ISL_12028893	EPI2017734	EPI2017735	EPI2017733	EPI2017737	EPI2017730	EPI2017736	EPI2017732	EPI2017731	A/turkey/Italy/21VIR11507/2021	*	H5N1
EPI_ISL_11007548	EPI1995225	EPI1995226	EPI1995224	EPI1995228	EPI1995221	EPI1995227	EPI1995223	EPI1995222	A/turkey/Italy/21VIR9520/2021		H5N1

Isolate_Id	PB2	PB1	PA	HA	NP	NA	MP	NS	Isolate_Name	Present in the phylogenetic network	Subtype
EPI_ISL_8882201	EPI1962054	EPI1962055	EPI1962053	EPI1962057	EPI1962050	EPI1962056	EPI1962052	EPI1962051	A/turkey/Italy/21VIR11803-1/2021		H5N1
EPI_ISL_8882187	EPI1962046	EPI1962047	EPI1962045	EPI1962049	EPI1962042	EPI1962048	EPI1962044	EPI1962043	A/turkey/Italy/21VIR11053-1/2021		H5N1
EPI_ISL_8882172	EPI1962030	EPI1962031	EPI1962029	EPI1962033	EPI1962026	EPI1962032	EPI1962028	EPI1962027	A/turkey/Italy/21VIR10851/2021		H5N1
EPI_ISL_8882171	EPI1962022	EPI1962023	EPI1962021	EPI1962025	EPI1962018	EPI1962024	EPI1962020	EPI1962019	A/turkey/Italy/21VIR11591-8/2021		H5N1
EPI_ISL_8882170	EPI1962014	EPI1962015	EPI1962013	EPI1962017	EPI1962010	EPI1962016	EPI1962012	EPI1962011	A/turkey/Italy/21VIR11887-3/2021	*	H5N1
EPI_ISL_8882167	EPI1961990	EPI1961991	EPI1961989	EPI1961993	EPI1961986	EPI1961992	EPI1961988	EPI1961987	A/turkey/Italy/21VIR11804-1/2021	*	H5N1
EPI_ISL_8882166	EPI1961982	EPI1961983	EPI1961981	EPI1961985	EPI1961978	EPI1961984	EPI1961980	EPI1961979	A/turkey/Italy/21VIR11586-2/2021		H5N1
EPI_ISL_14761272	EPI2141634	EPI2141635	EPI2141633	EPI2141638	EPI2141630	EPI2141636	EPI2141632	EPI2141631	A/turkey/Italy/IZSLT_22VIR366-3/2022		H5N1
EPI_ISL_7733598	EPI1944293	EPI1944294	EPI1944292	EPI1944296	EPI1944289	EPI1944295	EPI1944291	EPI1944290	A/turkey/Italy/21VIR9816-1/2021	*	H5N1
EPI_ISL_7733597	EPI1944285	EPI1944286	EPI1944284	EPI1944288	EPI1944281	EPI1944287	EPI1944283	EPI1944282	A/turkey/Italy/21VIR10340/2021	*	H5N1
EPI_ISL_7733587	EPI1944261	EPI1944262	EPI1944260	EPI1944264	EPI1944257	EPI1944263	EPI1944259	EPI1944258	A/turkey/Italy/21VIR9618-7/2021		H5N1
EPI_ISL_7733643	EPI1944466	EPI1944467	EPI1944465	EPI1944469	EPI1944462	EPI1944468	EPI1944464	EPI1944463	A/turkey/Italy/21VIR9649-2/2021	*	H5N1
EPI_ISL_7733638	EPI1944442	EPI1944443	EPI1944441	EPI1944445	EPI1944438	EPI1944444	EPI1944440	EPI1944439	A/turkey/Italy/21VIR9768-8/2021	*	H5N1
EPI_ISL_7733635	EPI1944434	EPI1944435	EPI1944433	EPI1944437	EPI1944430	EPI1944436	EPI1944432	EPI1944431	A/turkey/Italy/21VIR9510-1/2021	*	H5N1
EPI_ISL_7733633	EPI1944418	EPI1944419	EPI1944417	EPI1944421	EPI1944414	EPI1944420	EPI1944416	EPI1944415	A/turkey/Italy/21VIR9143-2/2021	*	H5N1
EPI_ISL_7733632	EPI1944410	EPI1944411	EPI1944409	EPI1944413	EPI1944406	EPI1944412	EPI1944408	EPI1944407	A/turkey/Italy/21VIR9512-1/2021	*	H5N1
EPI_ISL_7733629	EPI1944394	EPI1944395	EPI1944393	EPI1944397	EPI1944390	EPI1944396	EPI1944392	EPI1944391	A/turkey/Italy/21VIR9652-2/2021	*	H5N1
EPI_ISL_7733628	EPI1944386	EPI1944387	EPI1944385	EPI1944389	EPI1944382	EPI1944388	EPI1944384	EPI1944383	A/turkey/Italy/21VIR10251/2021		H5N1
EPI_ISL_7733624	EPI1944378	EPI1944379	EPI1944377	EPI1944381	EPI1944374	EPI1944380	EPI1944376	EPI1944375	A/turkey/Italy/21VIR9767-3/2021	*	H5N1
EPI_ISL_7733613	EPI1944354	EPI1944355	EPI1944353	EPI1944357	EPI1944350	EPI1944356	EPI1944352	EPI1944351	A/turkey/Italy/21VIR10456/2021	*	H5N1
EPI_ISL_5524384	EPI1923189	EPI1923190	EPI1923191	EPI1923192	EPI1923193	EPI1923194	EPI1923195	EPI1923196	A/turkey/Italy/21VIR8585-1/2021	*	H5N1
EPI_ISL_7733648	EPI1944498	EPI1944499	EPI1944497	EPI1944501	EPI1944494	EPI1944500	EPI1944496	EPI1944495	A/turkey/Italy/21VIR8817-1/2021	*	H5N1
EPI_ISL_7733647	EPI1944490	EPI1944491	EPI1944489	EPI1944493	EPI1944486	EPI1944492	EPI1944488	EPI1944487	A/turkey/Italy/21VIR9210-1/2021	*	H5N1
EPI_ISL_14761473	EPI2141802	EPI2141803	EPI2141800	EPI2141805	EPI2141797	EPI2141804	EPI2141799	EPI2141798	A/peacock/Italy/22VIR1953-2/2022		H5N1
EPI_ISL_14761439	EPI2141775	EPI2141776	EPI2141774	EPI2141779	EPI2141771	EPI2141777	EPI2141773	EPI2141772	A/laying_hen/Italy/22VIR1521-2/2022		H5N1
EPI_ISL_14761395	EPI2141740	EPI2141741	EPI2141739	EPI2141743	EPI2141736	EPI2141742	EPI2141738	EPI2141737	A/herring_gull/Italy/22VIR1710-3/2022		H5N1
EPI_ISL_14761373	EPI2141722	EPI2141723	EPI2141721	EPI2141726	EPI2141718	EPI2141724	EPI2141720	EPI2141719	A/buzzard/Italy/22VIR767-2/2022		H5N1
EPI_ISL_14761361	EPI2141713	EPI2141714	EPI2141712	EPI2141717	EPI2141709	EPI2141716	EPI2141711	EPI2141710	A/buzzard/Italy/22VIR767-1/2022		H5N1
EPI_ISL_14761349	EPI2141705	EPI2141706	EPI2141703	EPI2141708	EPI2141700	EPI2141707	EPI2141702	EPI2141701	A/hawk/Italy/22VIR428-7/2022		H5N1
EPI_ISL_14761338	EPI2141696	EPI2141697	EPI2141695	EPI2141699	EPI2141691	EPI2141698	EPI2141694	EPI2141693	A/hawk/Italy/22VIR428-5/2022		H5N1
EPI_ISL_14761318	EPI2141678	EPI2141679	EPI2141677	EPI2141682	EPI2141674	EPI2141681	EPI2141676	EPI2141675	A/laying_hen/Italy/22VIR204-1/2022		H5N1
EPI_ISL_14761302	EPI2141661	EPI2141662	EPI2141660	EPI2141664	EPI2141656	EPI2141663	EPI2141658	EPI2141657	A/laying_hen/Italy/22VIR203-3/2022		H5N1
EPI_ISL_14761261	EPI2141625	EPI2141626	EPI2141624	EPI2141629	EPI2141621	EPI2141627	EPI2141623	EPI2141622	A/laying_hen/Italy/22VIR48-1/2022		H5N1
EPI_ISL_14761176	EPI2141537	EPI2141538	EPI2141536	EPI2141540	EPI2141533	EPI2141539	EPI2141535	EPI2141534	A/laying_hen/Italy/21VIR11801-7/2021	*	H5N1
EPI_ISL_14761075	EPI2141458	EPI2141459	EPI2141457	EPI2141461	EPI2141453	EPI2141460	EPI2141456	EPI2141455	A/laying_hen/Italy/21VIR11592-1/2021		H5N1
EPI_ISL_14761065	EPI2141449	EPI2141450	EPI2141448	EPI2141452	EPI2141445	EPI2141451	EPI2141447	EPI2141446	A/laying_hen/Italy/21VIR11597-1/2021	*	H5N1
EPI_ISL_14761052	EPI2141440	EPI2141441	EPI2141439	EPI2141444	EPI2141436	EPI2141442	EPI2141438	EPI2141437	A/laying_hen/Italy/21VIR11595-5/2021	*	H5N1

Isolate_Id	PB2	PB1	PA	HA	NP	NA	MP	NS	Isolate_Name	Present in the phylogenetic network	Subtype
EPI_ISL_14760946	EPI2141352	EPI2141353	EPI2141351	EPI2141356	EPI2141348	EPI2141354	EPI2141350	EPI2141349	A/laying_hen/Italy/21VIR11498-1/2021		H5N1
EPI_ISL_14760936	EPI2141343	EPI2141344	EPI2141342	EPI2141347	EPI2141339	EPI2141345	EPI2141341	EPI2141340	A/laying_hen/Italy/21VIR11505-2/2021		H5N1
EPI_ISL_14760826	EPI2141256	EPI2141257	EPI2141255	EPI2141260	EPI2141252	EPI2141259	EPI2141254	EPI2141253	A/laying_hen/Italy/21VIR11486/2021		H5N1
EPI_ISL_14760803	EPI2141239	EPI2141240	EPI2141238	EPI2141242	EPI2141235	EPI2141241	EPI2141237	EPI2141236	A/laying_hen/Italy/21VIR11395-1/2021		H5N1
EPI_ISL_14760793	EPI2141230	EPI2141231	EPI2141229	EPI2141234	EPI2141226	EPI2141232	EPI2141228	EPI2141227	A/laying_hen/Italy/21VIR11394-1/2021		H5N1
EPI_ISL_14760755	EPI2141169	EPI2141170	EPI2141168	EPI2141173	EPI2141165	EPI2141172	EPI2141167	EPI2141166	A/laying_hen/Italy/21VIR11390-2/2021		H5N1
EPI_ISL_14760751	EPI2141135	EPI2141136	EPI2141134	EPI2141138	EPI2141130	EPI2141137	EPI2141133	EPI2141131	A/laying_hen/Italy/21VIR11299/2021	*	H5N1
EPI_ISL_14760750	EPI2141126	EPI2141127	EPI2141125	EPI2141129	EPI2141122	EPI2141128	EPI2141124	EPI2141123	A/laying_hen/Italy/21VIR11253-1/2021		H5N1
EPI_ISL_14760735	EPI2140996	EPI2140997	EPI2140995	EPI2140999	EPI2140991	EPI2140998	EPI2140994	EPI2140992	A/laying_hen/Italy/21VIR11300/2021	*	H5N1
EPI_ISL_14760733	EPI2140978	EPI2140979	EPI2140977	EPI2140982	EPI2140974	EPI2140980	EPI2140976	EPI2140975	A/laying_hen/Italy/21VIR11169/2021	*	H5N1
EPI_ISL_14760732	EPI2140969	EPI2140971	EPI2140968	EPI2140973	EPI2140965	EPI2140972	EPI2140967	EPI2140966	A/laying_hen/Italy/21VIR11168/2021	*	H5N1
EPI_ISL_14760731	EPI2140961	EPI2140962	EPI2140960	EPI2140964	EPI2140957	EPI2140963	EPI2140959	EPI2140958	A/laying_hen/Italy/21VIR11167/2021	*	H5N1
EPI_ISL_14760639	EPI2140160	EPI2140161	EPI2140159	EPI2140164	EPI2140156	EPI2140162	EPI2140158	EPI2140157	A/quail/Italy/21VIR10356/2021	*	H5N1
EPI_ISL_14760575	EPI2139645	EPI2139646	EPI2139644	EPI2139648	EPI2139641	EPI2139647	EPI2139643	EPI2139642	A/quail/Italy/21VIR9515-1/2021	*	H5N1
EPI_ISL_14760561	EPI2139533	EPI2139534	EPI2139532	EPI2139536	EPI2139529	EPI2139535	EPI2139531	EPI2139530	A/quail/Italy/21VIR9474-1/2021	*	H5N1
EPI_ISL_8882218	EPI1962131	EPI1962132	EPI1962130	EPI1962134	EPI1962127	EPI1962133	EPI1962129	EPI1962128	A/seagull/Italy/21VIR11259-12/2021		H5N1
EPI_ISL_8882216	EPI1962118	EPI1962119	EPI1962117	EPI1962121	EPI1962114	EPI1962120	EPI1962116	EPI1962115	A/buzzard/Italy/21VIR11899-5/2021		H5N1
EPI_ISL_8882215	EPI1962110	EPI1962111	EPI1962109	EPI1962113	EPI1962106	EPI1962112	EPI1962108	EPI1962107	A/owl/Italy/21VIR11899-1/2021		H5N1
EPI_ISL_8882214	EPI1962102	EPI1962103	EPI1962101	EPI1962105	EPI1962098	EPI1962104	EPI1962100	EPI1962099	A/heron/Italy/21VIR10998-1/2021		H5N1
EPI_ISL_8882213	EPI1962094	EPI1962095	EPI1962093	EPI1962097	EPI1962090	EPI1962096	EPI1962092	EPI1962091	A/seagull/Italy/21VIR11259-10/2021		H5N1
EPI_ISL_8882212	EPI1962086	EPI1962087	EPI1962085	EPI1962089	EPI1962082	EPI1962088	EPI1962084	EPI1962083	A/cignus_olor/Italy/IZSLT_21VIR10529-1/2021		H5N1
EPI_ISL_8882210	EPI1962070	EPI1962071	EPI1962069	EPI1962073	EPI1962066	EPI1962072	EPI1962068	EPI1962067	A/seagull/Italy/21VIR10481-2/2021	*	H5N1
EPI_ISL_8882209	EPI1962062	EPI1962063	EPI1962061	EPI1962065	EPI1962058	EPI1962064	EPI1962060	EPI1962059	A/little_owl/Italy/21VIR11382-1/2021	*	H5N1
EPI_ISL_8882173	EPI1962038	EPI1962039	EPI1962037	EPI1962041	EPI1962034	EPI1962040	EPI1962036	EPI1962035	A/laying_hen/Italy/21VIR11502/2021		H5N1
EPI_ISL_14761208	EPI2141563	EPI2141565	EPI2141562	EPI2141567	EPI2141559	EPI2141566	EPI2141561	EPI2141560	A/avian/Italy/21VIR11773-4/2021		H5N1
EPI_ISL_14760752	EPI2141143	EPI2141144	EPI2141142	EPI2141147	EPI2141139	EPI2141146	EPI2141141	EPI2141140	A/avian/Italy/21VIR11301/2021	*	H5N1
EPI_ISL_14760742	EPI2141057	EPI2141058	EPI2141055	EPI2141060	EPI2141052	EPI2141059	EPI2141054	EPI2141053	A/avian/Italy/21VIR11236/2021	*	H5N1
EPI_ISL_14760713	EPI2140804	EPI2140805	EPI2140803	EPI2140808	EPI2140800	EPI2140806	EPI2140802	EPI2140801	A/avian/Italy/21VIR10913-3/2021	*	H5N1
EPI_ISL_14760559	EPI2139517	EPI2139518	EPI2139516	EPI2139520	EPI2139513	EPI2139519	EPI2139515	EPI2139514	A/avian/Italy/21VIR9425-2/2021	*	H5N1
EPI_ISL_14761507	EPI2141828	EPI2141829	EPI2141827	EPI2141832	EPI2141824	EPI2141830	EPI2141826	EPI2141825	A/broiler/Italy/22VIR1892-3/2022		H5N1
EPI_ISL_14761496	EPI2141819	EPI2141820	EPI2141818	EPI2141823	EPI2141815	EPI2141821	EPI2141817	EPI2141816	A/chicken/Italy/22VIR1953-4/2022		H5N1
EPI_ISL_14761485	EPI2141810	EPI2141811	EPI2141809	EPI2141814	EPI2141806	EPI2141813	EPI2141808	EPI2141807	A/chicken/Italy/22VIR1953-3/2022		H5N1
EPI_ISL_14761451	EPI2141784	EPI2141785	EPI2141783	EPI2141787	EPI2141780	EPI2141786	EPI2141782	EPI2141781	A/chicken/Italy/22VIR1694-5/2022		H5N1
EPI_ISL_14761327	EPI2141687	EPI2141688	EPI2141686	EPI2141690	EPI2141683	EPI2141689	EPI2141685	EPI2141684	A/broiler/Italy/22VIR278-3/2022		H5N1
EPI_ISL_14761312	EPI2141669	EPI2141670	EPI2141668	EPI2141673	EPI2141665	EPI2141672	EPI2141667	EPI2141666	A/chicken/Italy/22VIR234-1/2022		H5N1
EPI_ISL_14761251	EPI2141616	EPI2141617	EPI2141615	EPI2141620	EPI2141612	EPI2141619	EPI2141614	EPI2141613	A/broiler/Italy/22VIR54-1/2022		H5N1
EPI_ISL_14761250	EPI2141608	EPI2141609	EPI2141606	EPI2141611	EPI2141603	EPI2141610	EPI2141605	EPI2141604	A/heron/Italy/22VIR1562/2022		H5N1

Isolate_Id	PB2	PB1	PA	HA	NP	NA	MP	NS	Isolate_Name	Present in the phylogenetic network	Subtype
EPI_ISL_14761249	EPI2141599	EPI2141600	EPI2141598	EPI2141602	EPI2141595	EPI2141601	EPI2141597	EPI2141596	A/broiler/Italy/21VIR11886-1/2021	*	H5N1
EPI_ISL_14761243	EPI2141590	EPI2141591	EPI2141589	EPI2141594	EPI2141586	EPI2141592	EPI2141588	EPI2141587	A/broiler/Italy/21VIR11847-1/2021	*	H5N1
EPI_ISL_14761220	EPI2141572	EPI2141573	EPI2141571	EPI2141576	EPI2141568	EPI2141575	EPI2141570	EPI2141569	A/chicken/Italy/21VIR11774-2/2021		H5N1
EPI_ISL_14761188	EPI2141546	EPI2141547	EPI2141545	EPI2141549	EPI2141542	EPI2141548	EPI2141544	EPI2141543	A/broiler/Italy/21VIR11802-1/2021	*	H5N1
EPI_ISL_14761165	EPI2141528	EPI2141529	EPI2141527	EPI2141532	EPI2141524	EPI2141530	EPI2141526	EPI2141525	A/broiler/Italy/21VIR11800/2021	*	H5N1
EPI_ISL_14761041	EPI2141431	EPI2141432	EPI2141430	EPI2141435	EPI2141427	EPI2141434	EPI2141429	EPI2141428	A/broiler/Italy/21VIR11583-1/2021	*	H5N1
EPI_ISL_14761020	EPI2141414	EPI2141415	EPI2141413	EPI2141417	EPI2141410	EPI2141416	EPI2141412	EPI2141411	A/chicken/Italy/21VIR11572/2021	*	H5N1
EPI_ISL_14761009	EPI2141405	EPI2141406	EPI2141404	EPI2141408	EPI2141401	EPI2141407	EPI2141403	EPI2141402	A/chicken/Italy/21VIR11571/2021	*	H5N1
EPI_ISL_14760880	EPI2141300	EPI2141301	EPI2141299	EPI2141303	EPI2141296	EPI2141302	EPI2141298	EPI2141297	A/chicken/Italy/21VIR11508/2021	*	H5N1
EPI_ISL_14760837	EPI2141265	EPI2141266	EPI2141264	EPI2141268	EPI2141261	EPI2141267	EPI2141263	EPI2141262	A/chicken/Italy/21VIR11485/2021	*	H5N1
EPI_ISL_14760774	EPI2141213	EPI2141214	EPI2141212	EPI2141216	EPI2141209	EPI2141215	EPI2141211	EPI2141210	A/chicken/Italy/21VIR11385-1/2021	*	H5N1
EPI_ISL_14760744	EPI2141074	EPI2141075	EPI2141073	EPI2141077	EPI2141070	EPI2141076	EPI2141072	EPI2141071	A/chicken/Italy/21VIR11238/2021		H5N1
EPI_ISL_14760741	EPI2141048	EPI2141049	EPI2141047	EPI2141051	EPI2141044	EPI2141050	EPI2141046	EPI2141045	A/chicken/Italy/21VIR11235/2021		H5N1
EPI_ISL_14760738	EPI2141022	EPI2141023	EPI2141021	EPI2141025	EPI2141018	EPI2141024	EPI2141020	EPI2141019	A/chicken/Italy/21VIR11056-6/2021		H5N1
EPI_ISL_14760737	EPI2141013	EPI2141014	EPI2141012	EPI2141016	EPI2141009	EPI2141015	EPI2141011	EPI2141010	A/chicken/Italy/21VIR11056-1/2021		H5N1
EPI_ISL_14760736	EPI2141004	EPI2141005	EPI2141003	EPI2141008	EPI2141000	EPI2141007	EPI2141002	EPI2141001	A/chicken/Italy/21VIR11054-1/2021		H5N1
EPI_ISL_14760734	EPI2140987	EPI2140988	EPI2140986	EPI2140990	EPI2140983	EPI2140989	EPI2140985	EPI2140984	A/chicken/Italy/21VIR11234/2021	*	H5N1
EPI_ISL_14760727	EPI2140926	EPI2140927	EPI2140925	EPI2140929	EPI2140922	EPI2140928	EPI2140924	EPI2140923	A/chicken/Italy/21VIR11055-1/2021		H5N1
EPI_ISL_14760723	EPI2140891	EPI2140892	EPI2140890	EPI2140895	EPI2140887	EPI2140893	EPI2140889	EPI2140888	A/chicken/Italy/21VIR10997-1/2021	*	H5N1
EPI_ISL_14760722	EPI2140883	EPI2140884	EPI2140881	EPI2140886	EPI2140878	EPI2140885	EPI2140880	EPI2140879	A/chicken/Italy/21VIR10999-1/2021	*	H5N1
EPI_ISL_14760721	EPI2140874	EPI2140875	EPI2140873	EPI2140877	EPI2140870	EPI2140876	EPI2140872	EPI2140871	A/chicken/Italy/21VIR10995-1/2021		H5N1
EPI_ISL_14760719	EPI2140856	EPI2140858	EPI2140855	EPI2140860	EPI2140852	EPI2140859	EPI2140854	EPI2140853	A/chicken/Italy/21VIR11075-2/2021	*	H5N1
EPI_ISL_14760718	EPI2140848	EPI2140849	EPI2140847	EPI2140851	EPI2140843	EPI2140850	EPI2140846	EPI2140845	A/chicken/Italy/21VIR11073-1/2021	*	H5N1
EPI_ISL_14760717	EPI2140839	EPI2140840	EPI2140838	EPI2140842	EPI2140835	EPI2140841	EPI2140837	EPI2140836	A/chicken/Italy/21VIR11078-10/2021	*	H5N1
EPI_ISL_14760716	EPI2140830	EPI2140831	EPI2140829	EPI2140834	EPI2140826	EPI2140833	EPI2140828	EPI2140827	A/chicken/Italy/21VIR11079-11/2021	*	H5N1
EPI_ISL_14760715	EPI2140822	EPI2140823	EPI2140821	EPI2140825	EPI2140817	EPI2140824	EPI2140819	EPI2140818	A/chicken/Italy/21VIR11076-1/2021	*	H5N1
EPI_ISL_14760714	EPI2140813	EPI2140814	EPI2140812	EPI2140816	EPI2140809	EPI2140815	EPI2140811	EPI2140810	A/chicken/Italy/21VIR10987-1/2021	*	H5N1
EPI_ISL_14760712	EPI2140795	EPI2140797	EPI2140794	EPI2140799	EPI2140791	EPI2140798	EPI2140793	EPI2140792	A/chicken/Italy/21VIR11071-2/2021	*	H5N1
EPI_ISL_14760711	EPI2140787	EPI2140788	EPI2140786	EPI2140790	EPI2140783	EPI2140789	EPI2140785	EPI2140784	A/chicken/Italy/21VIR11074-1/2021	*	H5N1
EPI_ISL_14760710	EPI2140778	EPI2140779	EPI2140777	EPI2140782	EPI2140774	EPI2140780	EPI2140776	EPI2140775	A/chicken/Italy/21VIR11072-1/2021	*	H5N1
EPI_ISL_14760709	EPI2140769	EPI2140770	EPI2140768	EPI2140773	EPI2140765	EPI2140772	EPI2140767	EPI2140766	A/chicken/Italy/21VIR10989/2021	*	H5N1
EPI_ISL_14760707	EPI2140752	EPI2140753	EPI2140751	EPI2140755	EPI2140748	EPI2140754	EPI2140750	EPI2140749	A/chicken/Italy/21VIR10969-3/2021		H5N1
EPI_ISL_14760706	EPI2140743	EPI2140744	EPI2140742	EPI2140747	EPI2140739	EPI2140745	EPI2140741	EPI2140740	A/chicken/Italy/21VIR10972-3/2021		H5N1
EPI_ISL_14760705	EPI2140735	EPI2140736	EPI2140734	EPI2140738	EPI2140730	EPI2140737	EPI2140733	EPI2140731	A/chicken/Italy/21VIR10973-4/2021		H5N1
EPI_ISL_14760704	EPI2140726	EPI2140727	EPI2140725	EPI2140729	EPI2140722	EPI2140728	EPI2140724	EPI2140723	A/chicken/Italy/21VIR10971-3/2021		H5N1
EPI_ISL_14760701	EPI2140700	EPI2140701	EPI2140699	EPI2140703	EPI2140696	EPI2140702	EPI2140698	EPI2140697	A/chicken/Italy/21VIR10843-1/2021		H5N1
EPI_ISL_14760698	EPI2140674	EPI2140675	EPI2140673	EPI2140677	EPI2140670	EPI2140676	EPI2140672	EPI2140671	A/chicken/Italy/21VIR10928/2021	*	H5N1

Isolate_Id	PB2	PB1	PA	HA	NP	NA	MP	NS	Isolate_Name	Present in the phylogenetic network	Subtype
EPI_ISL_14760696	EPI2140656	EPI2140657	EPI2140655	EPI2140660	EPI2140652	EPI2140658	EPI2140654	EPI2140653	A/chicken/Italy/21VIR10812-11/2021		H5N1
EPI_ISL_14760695	EPI2140647	EPI2140648	EPI2140646	EPI2140651	EPI2140643	EPI2140650	EPI2140645	EPI2140644	A/chicken/Italy/21VIR10811-28/2021		H5N1
EPI_ISL_14760692	EPI2140621	EPI2140622	EPI2140620	EPI2140624	EPI2140616	EPI2140623	EPI2140619	EPI2140618	A/chicken/Italy/21VIR10808-1/2021		H5N1
EPI_ISL_14760691	EPI2140612	EPI2140613	EPI2140611	EPI2140615	EPI2140608	EPI2140614	EPI2140610	EPI2140609	A/chicken/Italy/21VIR10814-1/2021		H5N1
EPI_ISL_14760688	EPI2140585	EPI2140586	EPI2140584	EPI2140589	EPI2140581	EPI2140588	EPI2140583	EPI2140582	A/chicken/Italy/21VIR10696-7/2021	*	H5N1
EPI_ISL_14760681	EPI2140523	EPI2140524	EPI2140522	EPI2140527	EPI2140519	EPI2140526	EPI2140521	EPI2140520	A/chicken/Italy/21VIR10853/2021		H5N1
EPI_ISL_14760678	EPI2140497	EPI2140498	EPI2140496	EPI2140500	EPI2140493	EPI2140499	EPI2140495	EPI2140494	A/chicken/Italy/21VIR10637-2/2021	*	H5N1
EPI_ISL_14760677	EPI2140488	EPI2140489	EPI2140487	EPI2140491	EPI2140484	EPI2140490	EPI2140486	EPI2140485	A/chicken/Italy/21VIR10635-1/2021		H5N1
EPI_ISL_14760676	EPI2140479	EPI2140480	EPI2140478	EPI2140483	EPI2140475	EPI2140481	EPI2140477	EPI2140476	A/chicken/Italy/21VIR10634-1/2021		H5N1
EPI_ISL_14760675	EPI2140470	EPI2140471	EPI2140469	EPI2140474	EPI2140466	EPI2140472	EPI2140468	EPI2140467	A/chicken/Italy/21VIR10723-1/2021	*	H5N1
EPI_ISL_14760674	EPI2140461	EPI2140463	EPI2140460	EPI2140465	EPI2140457	EPI2140464	EPI2140459	EPI2140458	A/chicken/Italy/21VIR10724-13/2021	*	H5N1
EPI_ISL_14760673	EPI2140453	EPI2140454	EPI2140451	EPI2140456	EPI2140448	EPI2140455	EPI2140450	EPI2140449	A/chicken/Italy/21VIR10620-1/2021	*	H5N1
EPI_ISL_14760672	EPI2140444	EPI2140445	EPI2140443	EPI2140447	EPI2140439	EPI2140446	EPI2140441	EPI2140440	A/chicken/Italy/21VIR10619-1/2021	*	H5N1
EPI_ISL_14760671	EPI2140435	EPI2140436	EPI2140434	EPI2140438	EPI2140430	EPI2140437	EPI2140433	EPI2140432	A/chicken/Italy/21VIR10618-19/2021	*	H5N1
EPI_ISL_14760670	EPI2140426	EPI2140427	EPI2140425	EPI2140429	EPI2140422	EPI2140428	EPI2140424	EPI2140423	A/chicken/Italy/21VIR10616-1/2021	*	H5N1
EPI_ISL_14760666	EPI2140390	EPI2140392	EPI2140389	EPI2140394	EPI2140386	EPI2140393	EPI2140388	EPI2140387	A/chicken/Italy/21VIR10522-13/2021	*	H5N1
EPI_ISL_14760665	EPI2140382	EPI2140383	EPI2140381	EPI2140385	EPI2140377	EPI2140384	EPI2140379	EPI2140378	A/chicken/Italy/21VIR10463-1/2021	*	H5N1
EPI_ISL_14760661	EPI2140346	EPI2140347	EPI2140345	EPI2140350	EPI2140342	EPI2140348	EPI2140344	EPI2140343	A/chicken/Italy/21VIR10386/2021	*	H5N1
EPI_ISL_14760659	EPI2140328	EPI2140330	EPI2140327	EPI2140332	EPI2140324	EPI2140331	EPI2140326	EPI2140325	A/chicken/Italy/21VIR10469/2021	*	H5N1
EPI_ISL_14760658	EPI2140320	EPI2140321	EPI2140319	EPI2140323	EPI2140315	EPI2140322	EPI2140317	EPI2140316	A/chicken/Italy/21VIR10385/2021	*	H5N1
EPI_ISL_14760655	EPI2140302	EPI2140303	EPI2140301	EPI2140305	EPI2140298	EPI2140304	EPI2140300	EPI2140299	A/chicken/Italy/21VIR10387/2021	*	H5N1
EPI_ISL_14760654	EPI2140293	EPI2140294	EPI2140292	EPI2140297	EPI2140289	EPI2140295	EPI2140291	EPI2140290	A/chicken/Italy/21VIR10470/2021	*	H5N1
EPI_ISL_14760651	EPI2140266	EPI2140268	EPI2140265	EPI2140270	EPI2140262	EPI2140269	EPI2140264	EPI2140263	A/chicken/Italy/21VIR10394/2021	*	H5N1
EPI_ISL_14760648	EPI2140240	EPI2140241	EPI2140239	EPI2140243	EPI2140236	EPI2140242	EPI2140238	EPI2140237	A/chicken/Italy/21VIR10382/2021	*	H5N1
EPI_ISL_14760646	EPI2140222	EPI2140223	EPI2140221	EPI2140226	EPI2140218	EPI2140224	EPI2140220	EPI2140219	A/chicken/Italy/21VIR10265/2021	*	H5N1
EPI_ISL_14760645	EPI2140213	EPI2140214	EPI2140212	EPI2140217	EPI2140209	EPI2140215	EPI2140211	EPI2140210	A/chicken/Italy/21VIR10264/2021	*	H5N1
EPI_ISL_14760644	EPI2140204	EPI2140205	EPI2140203	EPI2140208	EPI2140200	EPI2140207	EPI2140202	EPI2140201	A/chicken/Italy/21VIR10271/2021	*	H5N1
EPI_ISL_14760643	EPI2140195	EPI2140197	EPI2140194	EPI2140199	EPI2140191	EPI2140198	EPI2140193	EPI2140192	A/chicken/Italy/21VIR10263/2021	*	H5N1
EPI_ISL_14760642	EPI2140187	EPI2140188	EPI2140185	EPI2140190	EPI2140182	EPI2140189	EPI2140184	EPI2140183	A/chicken/Italy/21VIR10262/2021	*	H5N1
EPI_ISL_14760641	EPI2140178	EPI2140179	EPI2140177	EPI2140181	EPI2140174	EPI2140180	EPI2140176	EPI2140175	A/chicken/Italy/21VIR10357/2021	*	H5N1
EPI_ISL_14760640	EPI2140169	EPI2140170	EPI2140168	EPI2140172	EPI2140165	EPI2140171	EPI2140167	EPI2140166	A/chicken/Italy/21VIR10358/2021	*	H5N1
EPI_ISL_14760636	EPI2140136	EPI2140137	EPI2140135	EPI2140139	EPI2140132	EPI2140138	EPI2140134	EPI2140133	A/chicken/Italy/21VIR10146/2021	*	H5N1
EPI_ISL_14760635	EPI2140128	EPI2140129	EPI2140127	EPI2140131	EPI2140124	EPI2140130	EPI2140126	EPI2140125	A/chicken/Italy/21VIR10252/2021	*	H5N1
EPI_ISL_14760629	EPI2140080	EPI2140081	EPI2140079	EPI2140083	EPI2140076	EPI2140082	EPI2140078	EPI2140077	A/chicken/Italy/21VIR10094/2021	*	H5N1
EPI_ISL_14760626	EPI2140054	EPI2140055	EPI2140053	EPI2140057	EPI2140050	EPI2140056	EPI2140052	EPI2140051	A/chicken/Italy/21VIR10093/2021	*	H5N1
EPI_ISL_14760625	EPI2140045	EPI2140047	EPI2140043	EPI2140049	EPI2140040	EPI2140048	EPI2140042	EPI2140041	A/chicken/Italy/21VIR10096/2021	*	H5N1
EPI_ISL_14760624	EPI2140036	EPI2140037	EPI2140035	EPI2140039	EPI2140032	EPI2140038	EPI2140034	EPI2140033	A/chicken/Italy/21VIR9940-5/2021	*	H5N1

Isolate_Id	PB2	PB1	PA	HA	NP	NA	MP	NS	Isolate_Name	Present in the phylogenetic network	Subtype
EPI_ISL_14760622	EPI2140020	EPI2140021	EPI2140019	EPI2140023	EPI2140016	EPI2140022	EPI2140018	EPI2140017	A/chicken/Italy/21VIR9944-2/2021	*	H5N1
EPI_ISL_14760620	EPI2140004	EPI2140005	EPI2140003	EPI2140007	EPI2140000	EPI2140006	EPI2140002	EPI2140001	A/chicken/Italy/21VIR9998/2021	*	H5N1
EPI_ISL_14760616	EPI2139972	EPI2139973	EPI2139971	EPI2139975	EPI2139968	EPI2139974	EPI2139970	EPI2139969	A/chicken/Italy/21VIR10000/2021	*	H5N1
EPI_ISL_14760612	EPI2139940	EPI2139941	EPI2139939	EPI2139943	EPI2139936	EPI2139942	EPI2139938	EPI2139937	A/chicken/Italy/21VIR9858-3/2021	*	H5N1
EPI_ISL_14760605	EPI2139885	EPI2139886	EPI2139884	EPI2139888	EPI2139881	EPI2139887	EPI2139883	EPI2139882	A/chicken/Italy/21VIR9904-18/2021		H5N1
EPI_ISL_14760604	EPI2139877	EPI2139878	EPI2139876	EPI2139880	EPI2139873	EPI2139879	EPI2139875	EPI2139874	A/chicken/Italy/21VIR9904-17/2021		H5N1
EPI_ISL_14760603	EPI2139869	EPI2139870	EPI2139868	EPI2139872	EPI2139865	EPI2139871	EPI2139867	EPI2139866	A/chicken/Italy/21VIR9904-16/2021	*	H5N1
EPI_ISL_14760602	EPI2139861	EPI2139862	EPI2139860	EPI2139864	EPI2139857	EPI2139863	EPI2139859	EPI2139858	A/chicken/Italy/21VIR9830-27/2021	*	H5N1
EPI_ISL_14760598	EPI2139829	EPI2139830	EPI2139828	EPI2139832	EPI2139825	EPI2139831	EPI2139827	EPI2139826	A/chicken/Italy/21VIR9902-2/2021	*	H5N1
EPI_ISL_14760597	EPI2139821	EPI2139822	EPI2139820	EPI2139824	EPI2139817	EPI2139823	EPI2139819	EPI2139818	A/chicken/Italy/21VIR9868-8/2021	*	H5N1
EPI_ISL_14760582	EPI2139701	EPI2139702	EPI2139700	EPI2139704	EPI2139697	EPI2139703	EPI2139699	EPI2139698	A/chicken/Italy/21VIR9580-23/2021		H5N1
EPI_ISL_14760568	EPI2139589	EPI2139590	EPI2139588	EPI2139592	EPI2139585	EPI2139591	EPI2139587	EPI2139586	A/chicken/Italy/21VIR9508-3/2021	*	H5N1
EPI_ISL_14760567	EPI2139581	EPI2139582	EPI2139580	EPI2139584	EPI2139577	EPI2139583	EPI2139579	EPI2139578	A/chicken/Italy/21VIR9507-7/2021	*	H5N1
EPI_ISL_14760566	EPI2139573	EPI2139574	EPI2139572	EPI2139576	EPI2139569	EPI2139575	EPI2139571	EPI2139570	A/chicken/Italy/21VIR9509-1/2021	*	H5N1
EPI_ISL_14760558	EPI2139509	EPI2139510	EPI2139508	EPI2139512	EPI2139505	EPI2139511	EPI2139507	EPI2139506	A/chicken/Italy/21VIR9371-1/2021	*	H5N1
EPI_ISL_14760557	EPI2139501	EPI2139502	EPI2139500	EPI2139504	EPI2139497	EPI2139503	EPI2139499	EPI2139498	A/chicken/Italy/21VIR9219-6/2021	*	H5N1
EPI_ISL_14760552	EPI2139461	EPI2139462	EPI2139460	EPI2139464	EPI2139457	EPI2139463	EPI2139459	EPI2139458	A/chicken/Italy/21VIR9212-1/2021	*	H5N1
EPI_ISL_14760550	EPI2139445	EPI2139446	EPI2139444	EPI2139448	EPI2139441	EPI2139447	EPI2139443	EPI2139442	A/chicken/Italy/21VIR9133-21/2021		H5N1
EPI_ISL_14760548	EPI2139429	EPI2139430	EPI2139428	EPI2139432	EPI2139425	EPI2139431	EPI2139427	EPI2139426	A/chicken/Italy/21VIR9074-10/2021	*	H5N1
EPI_ISL_8882169	EPI1962006	EPI1962007	EPI1962005	EPI1962009	EPI1962002	EPI1962008	EPI1962004	EPI1962003	A/chicken/Italy/21VIR10573-1/2021	*	H5N1
EPI_ISL_8882168	EPI1961998	EPI1961999	EPI1961997	EPI1962001	EPI1961994	EPI1962000	EPI1961996	EPI1961995	A/chicken/Italy/21VIR10850/2021	*	H5N1
EPI_ISL_14761519	EPI2141837	EPI2141838	EPI2141836	EPI2141840	EPI2141833	EPI2141839	EPI2141835	EPI2141834	A/chicken/Italy/IZSLT22VIR2562-1/2022		H5N1
EPI_ISL_7733603	EPI1944309	EPI1944310	EPI1944308	EPI1944312	EPI1944305	EPI1944311	EPI1944307	EPI1944306	A/chicken/Italy/21VIR9951-25/2021	*	H5N1
EPI_ISL_7733599	EPI1944301	EPI1944302	EPI1944300	EPI1944304	EPI1944297	EPI1944303	EPI1944299	EPI1944298	A/chicken/Italy/21VIR10389/2021		H5N1
EPI_ISL_7733595	EPI1944277	EPI1944278	EPI1944276	EPI1944280	EPI1944273	EPI1944279	EPI1944275	EPI1944274	A/chicken/Italy/21VIR10239/2021	*	H5N1
EPI_ISL_7733582	EPI1944237	EPI1944238	EPI1944236	EPI1944240	EPI1944233	EPI1944239	EPI1944235	EPI1944234	A/chicken/Italy/21VIR10388/2021		H5N1
EPI_ISL_7733641	EPI1944450	EPI1944451	EPI1944449	EPI1944453	EPI1944446	EPI1944452	EPI1944448	EPI1944447	A/chicken/Italy/21VIR10352/2021		H5N1
EPI_ISL_7733634	EPI1944426	EPI1944427	EPI1944425	EPI1944429	EPI1944422	EPI1944428	EPI1944424	EPI1944423	A/chicken/Italy/21VIR9765-12/2021	*	H5N1
EPI_ISL_7733630	EPI1944402	EPI1944403	EPI1944401	EPI1944405	EPI1944398	EPI1944404	EPI1944400	EPI1944399	A/chicken/Italy/21VIR9691-2/2021	*	H5N1
EPI_ISL_7733617	EPI1944362	EPI1944363	EPI1944361	EPI1944365	EPI1944358	EPI1944364	EPI1944360	EPI1944359	A/chicken/Italy/21VIR9133-20/2021	*	H5N1
EPI_ISL_7733609	EPI1944325	EPI1944326	EPI1944324	EPI1944328	EPI1944321	EPI1944327	EPI1944323	EPI1944322	A/chicken/Italy/21VIR9580-22/2021	*	H5N1
EPI_ISL_7733607	EPI1944317	EPI1944318	EPI1944316	EPI1944320	EPI1944313	EPI1944319	EPI1944315	EPI1944314	A/chicken/Italy/21VIR10384/2021	*	H5N1
EPI_ISL_7733644	EPI1944474	EPI1944475	EPI1944473	EPI1944477	EPI1944470	EPI1944476	EPI1944472	EPI1944471	A/chicken/Italy/IZSLT-122448_21VIR9218-1/2021		H5N1
EPI_ISL_14761417	EPI2141758	EPI2141759	EPI2141756	EPI2141761	EPI2141753	EPI2141760	EPI2141755	EPI2141754	A/duck/Italy/22VIR1295-1/2022		H5N1
EPI_ISL_14761407	EPI2141749	EPI2141750	EPI2141748	EPI2141752	EPI2141744	EPI2141751	EPI2141747	EPI2141746	A/duck/Italy/22VIR1294-1/2022		H5N1
EPI_ISL_14760988	EPI2141387	EPI2141388	EPI2141386	EPI2141391	EPI2141383	EPI2141390	EPI2141385	EPI2141384	A/duck/Italy/21VIR11501/2021		H5N1
EPI_ISL_14760693	EPI2140630	EPI2140631	EPI2140629	EPI2140633	EPI2140625	EPI2140632	EPI2140627	EPI2140626	A/duck/Italy/21VIR10809-1/2021		H5N1

Isolate_Id	PB2	PB1	PA	HA	NP	NA	MP	NS	Isolate_Name	Present in the phylogenetic network	Subtype
EPI_ISL_14760662	EPI2140355	EPI2140356	EPI2140354	EPI2140359	EPI2140351	EPI2140357	EPI2140353	EPI2140352	A/duck/Italy/21VIR10523-1/2021		H5N1
EPI_ISL_14760608	EPI2139909	EPI2139910	EPI2139908	EPI2139912	EPI2139905	EPI2139911	EPI2139907	EPI2139906	A/duck/Italy/21VIR9836-15/2021	*	H5N1
EPI_ISL_14760562	EPI2139541	EPI2139542	EPI2139540	EPI2139544	EPI2139537	EPI2139543	EPI2139539	EPI2139538	A/duck/Italy/21VIR9373-2/2021	*	H5N1
EPI_ISL_7733584	EPI1944245	EPI1944246	EPI1944244	EPI1944248	EPI1944241	EPI1944247	EPI1944243	EPI1944242	A/duck/Italy/21VIR10447/2021		H5N1
EPI_ISL_7733591	EPI1944269	EPI1944270	EPI1944268	EPI1944272	EPI1944265	EPI1944271	EPI1944267	EPI1944266	A/mallard/Italy/21VIR8919-2/2021		H5N1
EPI_ISL_7733585	EPI1944253	EPI1944254	EPI1944252	EPI1944256	EPI1944249	EPI1944255	EPI1944251	EPI1944250	A/Eurasian_wigeon/Italy/21VIR8919-3/2021		H5N1
EPI_ISL_7733642	EPI1944458	EPI1944459	EPI1944457	EPI1944461	EPI1944454	EPI1944460	EPI1944456	EPI1944455	A/kestrel/Italy/21VIR10468/2021	*	H5N1
EPI_ISL_7733621	EPI1944370	EPI1944371	EPI1944369	EPI1944373	EPI1944366	EPI1944372	EPI1944368	EPI1944367	A/seagull/Italy/21VIR9432-2/2021	*	H5N1
EPI_ISL_7733612	EPI1944346	EPI1944347	EPI1944345	EPI1944349	EPI1944342	EPI1944348	EPI1944344	EPI1944343	A/magpie/Italy/21VIR9487-2/2021		H5N1
EPI_ISL_7733611	EPI1944338	EPI1944339	EPI1944337	EPI1944341	EPI1944334	EPI1944340	EPI1944336	EPI1944335	A/Wild_goose/Italy/21VIR10193/2021		H5N1
