# Supplementary material for: Integration of Epidemiological and Genomic Data to Investigate H5N1 HPAI Outbreaks in Northern Italy in 2021–2022
Source: Pathogens. 2023 Jan 6;12(1):100. doi: 10.3390/pathogens12010100 (PMC9865711; doi:10.3390/pathogens12010100)
Supplement: Supplementary file 1 [file pathogens-12-00100-s001.zip › Supplementary Table S2.pdf]

Supplementary Table S2. Information from the GISAID EpiFlu database on hemagglutinin segments of the European viruses.

Segment ID	Country	Collection date	Isolate-ID	Isolate name	Originating Lab	Submitting Lab	Authors
EPI2014953	Poland	2022-Feb-05	EPI_ISL_11922811	A/goose/Poland/H124_22VIR2515-5/2022	National Veterinary Research Institute	Istituto Zooprofilattico Sperimentale delle Venezie	Swieton, E.; Smietanka, K.; Barbierato, G.; Zecchin, B.; Fusaro, A.; Schivo, A.; Salviato, A.; Palumbo, E.; Giussani, E.; Monne, I.; Terregino, C.
EPI1998426	Spain	2022-Jan-26	EPI_ISL_11259319	A/goose/Spain/294-2_22VIR2142-9/2022	Laboratorio Central de Veterinaria	Istituto Zooprofilattico Sperimentale delle Venezie	Ruano, M.J.; Rocha, A.; Sanchez, A.; Aguero, M; Barbierato, G.; Zecchin, B.; Fusaro, A.; Schivo, A.; Salviato, A.; Palumbo, E.; Giussani, E.; Monne, I.; Terregino, C.
EPI1998418	Spain	2022-Jan-22	EPI_ISL_11259318	A/goose/Spain/239-1_22VIR2142-8/2022	Laboratorio Central de Veterinaria	Istituto Zooprofilattico Sperimentale delle Venezie	Ruano, M.J.; Rocha, A.; Sanchez, A.; Aguero, M; Barbierato, G.; Zecchin, B.; Fusaro, A.; Schivo, A.; Salviato, A.; Palumbo, E.; Giussani, E.; Monne, I.; Terregino, C.
EPI1998402	Spain	2022-Jan-14	EPI_ISL_11259316	A/goose/Spain/141-9_22VIR2142-6/2022	Laboratorio Central de Veterinaria	Istituto Zooprofilattico Sperimentale delle Venezie	Ruano, M.J.; Rocha, A.; Sanchez, A.; Aguero, M; Barbierato, G.; Zecchin, B.; Fusaro, A.; Schivo, A.; Salviato, A.; Palumbo, E.; Giussani, E.; Monne, I.; Terregino, C.
EPI1998394	Spain	2022-Jan-10	EPI_ISL_11259315	A/goose/Spain/88-3_22VIR2142-4/2022	Laboratorio Central de Veterinaria	Istituto Zooprofilattico Sperimentale delle Venezie	Ruano, M.J.; Rocha, A.; Sanchez, A.; Aguero, M; Barbierato, G.; Zecchin, B.; Fusaro, A.; Schivo, A.; Salviato, A.; Palumbo, E.; Giussani, E.; Monne, I.; Terregino, C.
EPI1998298	Spain	2022-Jan-07	EPI_ISL_11259303	A/goose/Spain/65-3_22VIR2142-2/2022	Laboratorio Central de Veterinaria	Istituto Zooprofilattico Sperimentale delle Venezie	Ruano, M.J.; Rocha, A.; Sanchez, A.; Aguero, M; Barbierato, G.; Zecchin, B.; Fusaro, A.; Schivo, A.; Salviato, A.; Palumbo, E.; Giussani, E.; Monne, I.; Terregino, C.
EPI1998162	Spain	2022-Feb-04	EPI_ISL_11259286	A/goose/Spain/512-2_22VIR2142-13/2022	Laboratorio Central de Veterinaria	Istituto Zooprofilattico Sperimentale delle Venezie	Ruano, M.J.; Rocha, A.; Sanchez, A.; Aguero, M; Barbierato, G.; Zecchin, B.; Fusaro, A.; Schivo, A.; Salviato, A.; Palumbo, E.; Giussani, E.; Monne, I.; Terregino, C.
EPI1998146	Ireland	2022-Feb-14	EPI_ISL_11259284	A/goose/Ireland/3869_22VIR2064-2/2022	Central Veterinary Research Laboratory	Istituto Zooprofilattico Sperimentale delle Venezie	Byrne, C.; Garcia, K.; Cuartero, L.G.; Barbierato, G.; Zecchin, B.; Fusaro, A.; Schivo, A.; Salviato, A.; Palumbo, E.; Giussani, E.; Monne, I.; Terregino, C.
EPI1968921	France	2021-Dec-23	EPI_ISL_9377021	A/goose/France/21P014207/2021	Anses (Ploufragan-Plouzané)	ANSES Agence Nationale De Securite Sanitaire De L'alimentation	NA
EPI1949898	France	2021-Nov-25	EPI_ISL_8377418	A/goose/France/21P013228/2021	Anses (Ploufragan-Plouzané)	ANSES Agence Nationale De Securite Sanitaire De L'alimentation	NA
EPI1848934	United Kingdom	2021-Feb-10	EPI_ISL_1123361	A/pheasant/Scotland/000348/2021	Animal and Plant Health Agency (APHA)	Animal and Plant Health Agency (APHA)	NA

Segment ID	Country	Collection date	Isolate-ID	Isolate name	Originating Lab	Submitting Lab	Authors
EPI1966009	Belgium	2022-Jan-04	EPI_ISL_9162331	A/Phasianus_colchicus/Belgium/294/2022	Sciensano - Animal Infectious Diseases	Sciensano, Department of Animal Infectious Diseases	Van Borm, Steven; Roupie, Virginie; Lambrecht, Benedicte; Mathijs, Elisabeth; Steensels, Mieke
EPI2014977	Poland	2022-Feb-17	EPI_ISL_11922814	A/swan/Poland/MB083_22VIR2515-8/2022	National Veterinary Research Institute	Istituto Zooprofilattico Sperimentale delle Venezie	Swieton, E.; Smietanka, K.; Barbierato, G.; Zecchin, B.; Fusaro, A.; Schivo, A.; Salviato, A.; Palumbo, E.; Giussani, E.; Monne, I.; Terregino, C.
EPI2014969	Poland	2022-Feb-10	EPI_ISL_11922813	A/swan/Poland/MB078_22VIR2515-7/2022	National Veterinary Research Institute	Istituto Zooprofilattico Sperimentale delle Venezie	Swieton, E.; Smietanka, K.; Barbierato, G.; Zecchin, B.; Fusaro, A.; Schivo, A.; Salviato, A.; Palumbo, E.; Giussani, E.; Monne, I.; Terregino, C.
EPI1998210	Spain	2021-Dec-22	EPI_ISL_11259292	A/swan/Spain/4087-1_22VIR2142-1/2021	Laboratorio Central de Veterinaria	Istituto Zooprofilattico Sperimentale delle Venezie	Ruano, M.J.; Rocha, A.; Sanchez, A.; Aguero, M; Barbierato, G.; Zecchin, B.; Fusaro, A.; Schivo, A.; Salviato, A.; Palumbo, E.; Giussani, E.; Monne, I.; Terregino, C.
EPI1995124	Slovenia	2022-Jan-03	EPI_ISL_11007535	A/swan/Slovenia/13_22VIR777-8/2022	University of Ljubljana	Istituto Zooprofilattico Sperimentale delle Venezie	Slavec, B.; Ra?nik, J.; Krape?, U.; ?labravec, Z.; A?ko, J.; Cociancich, V.; Paller, T.; Vidrih, ?.; Rojs, O.Z.; Arseniev, S.; Groza, O.; Barbierato, G.; Zecchin, B.; Fusaro, A.; Schivo, A.; Salviato, A.; Palumbo, E.; Giussani, E.; Monne, I.; Terregino, C.
EPI1995108	Slovenia	2021-Dec-30	EPI_ISL_11007533	A/swan/Slovenia/2073_22VIR777-6/2021	University of Ljubljana	Istituto Zooprofilattico Sperimentale delle Venezie	Slavec, B.; Ra?nik, J.; Krape?, U.; ?labravec, Z.; A?ko, J.; Cociancich, V.; Paller, T.; Vidrih, ?.; Rojs, O.Z.; Arseniev, S.; Groza, O.; Barbierato, G.; Zecchin, B.; Fusaro, A.; Schivo, A.; Salviato, A.; Palumbo, E.; Giussani, E.; Monne, I.; Terregino, C.
EPI1995100	Slovenia	2021-Dec-30	EPI_ISL_11007532	A/swan/Slovenia/2072_22VIR777-5/2021	University of Ljubljana	Istituto Zooprofilattico Sperimentale delle Venezie	Slavec, B.; Ra?nik, J.; Krape?, U.; ?labravec, Z.; A?ko, J.; Cociancich, V.; Paller, T.; Vidrih, ?.; Rojs, O.Z.; Arseniev, S.; Groza, O.; Barbierato, G.; Zecchin, B.; Fusaro, A.; Schivo, A.; Salviato, A.; Palumbo, E.; Giussani, E.; Monne, I.; Terregino, C.
EPI1995092	Slovenia	2021-Dec-29	EPI_ISL_11007531	A/swan/Slovenia/2060_22VIR777-4/2021	University of Ljubljana	Istituto Zooprofilattico Sperimentale delle Venezie	Slavec, B.; Ra?nik, J.; Krape?, U.; ?labravec, Z.; A?ko, J.; Cociancich, V.; Paller, T.; Vidrih, ?.; Rojs, O.Z.; Arseniev, S.; Groza, O.; Barbierato, G.; Zecchin, B.; Fusaro, A.; Schivo, A.; Salviato, A.; Palumbo, E.; Giussani, E.; Monne, I.; Terregino, C.
EPI1995084	Slovenia	2021-Dec-27	EPI_ISL_11007530	A/swan/Slovenia/2049_22VIR777-3/2021	University of Ljubljana	Istituto Zooprofilattico Sperimentale delle Venezie	Slavec, B.; Ra?nik, J.; Krape?, U.; ?labravec, Z.; A?ko, J.; Cociancich, V.; Paller, T.; Vidrih, ?.; Rojs, O.Z.; Arseniev, S.; Groza, O.; Barbierato, G.; Zecchin, B.; Fusaro, A.; Schivo, A.; Salviato, A.; Palumbo, E.; Giussani, E.; Monne, I.; Terregino, C.
EPI1995076	Slovenia	2021-Dec-26	EPI_ISL_11007529	A/swan/Slovenia/2041_22VIR777-2/2021	University of Ljubljana	Istituto Zooprofilattico Sperimentale delle Venezie	Slavec, B.; Ra?nik, J.; Krape?, U.; ?labravec, Z.; A?ko, J.; Cociancich, V.; Paller, T.; Vidrih, ?.; Rojs, O.Z.; Arseniev, S.; Groza, O.; Barbierato, G.; Zecchin, B.; Fusaro, A.; Schivo, A.; Salviato, A.; Palumbo, E.; Giussani, E.; Monne, I.; Terregino, C.
EPI1840056	United Kingdom	2020-Dec-03	EPI_ISL_766876	A/mute_swan/England/234255/2020	Animal and Plant Health Agency (APHA)	Animal and Plant Health Agency (APHA)	NA
EPI1963897	United Kingdom	2021-Nov-09	EPI_ISL_9029962	A/Whooper_swan/Scotland/056219/2021	Animal and Plant Health Agency (APHA)	Animal and Plant Health Agency (APHA)	NA

Segment ID	Country	Collection date	Isolate-ID	Isolate name	Originating Lab	Submitting Lab	Authors
EPI1963881	United Kingdom	2021-Nov-11	EPI_ISL_9029960	A/mute_swan/England/385466/2021	Animal and Plant Health Agency (APHA)	Animal and Plant Health Agency (APHA)	NA
EPI1950887	Romania	2021-Nov-26	EPI_ISL_8440976	A/mute_swan/Romania/16790_21VIR11355/2021	Institute for Diagnosis & Animal Health (IDAH)	Istituto Zooprofilattico Sperimentale Delle Venezie	Burlacu, R.; Neicut, A.; Onita, I.; Motiu, R.; Zecchin, B.; Fusaro, A.; Schivo, A.; Salviato, A.; Palumbo, E.; Milani, A.; Giussani, E.; Pastori, A.; Monne, I.; Terregino, C.
EPI1950879	Romania	2021-Nov-11	EPI_ISL_8440175	A/Cygnus_olor/Romania/16381_21VIR10306/2021	Institute for Diagnosis & Animal Health (IDAH)	Istituto Zooprofilattico Sperimentale Delle Venezie	Burlacu, R.; Neicut, A.; Onita, I.; Motiu, R.; Zecchin, B.; Fusaro, A.; Schivo, A.; Salviato, A.; Palumbo, E.; Milani, A.; Giussani, E.; Pastori, A.; Monne, I.; Terregino, C.
EPI1937671	Poland	2021-Nov-08	EPI_ISL_6937114	A/mute_swan/Poland/MB490-L1/2021	National Veterinary Research Institut Poland, PIWet-PIB	National Veterinary Research Institut Poland, PIWet-PIB	E. Swieton, K. Smietanka
EPI1928148	France	2021-Nov-08	EPI_ISL_6590766	A/swan/France/21P012384/2021	Anses (Ploufragan-Plouzané)	ANSES Agence Nationale De Securite Sanitaire De L'alimentation	NA
EPI1924123	United Kingdom	2021-Oct-24	EPI_ISL_5804708	A/mute_swan/England/053054/2021	Animal and Plant Health Agency (APHA)	Animal and Plant Health Agency (APHA)	NA
EPI1998362	Spain	2022-Feb-15	EPI_ISL_11259311	A/turkey/Spain/646-7_22VIR2142-37/2022	Laboratorio Central de Veterinaria	Istituto Zooprofilattico Sperimentale delle Venezie	Ruano, M.J.; Rocha, A.; Sanchez, A.; Aguero, M; Barbierato, G.; Zecchin, B.; Fusaro, A.; Schivo, A.; Salviato, A.; Palumbo, E.; Giussani, E.; Monne, I.; Terregino, C.
EPI1998354	Spain	2022-Feb-15	EPI_ISL_11259310	A/turkey/Spain/645-1_22VIR2142-36/2022	Laboratorio Central de Veterinaria	Istituto Zooprofilattico Sperimentale delle Venezie	Ruano, M.J.; Rocha, A.; Sanchez, A.; Aguero, M; Barbierato, G.; Zecchin, B.; Fusaro, A.; Schivo, A.; Salviato, A.; Palumbo, E.; Giussani, E.; Monne, I.; Terregino, C.
EPI1998322	Spain	2022-Feb-14	EPI_ISL_11259306	A/turkey/Spain/586-4_22VIR2142-32/2022	Laboratorio Central de Veterinaria	Istituto Zooprofilattico Sperimentale delle Venezie	Ruano, M.J.; Rocha, A.; Sanchez, A.; Aguero, M; Barbierato, G.; Zecchin, B.; Fusaro, A.; Schivo, A.; Salviato, A.; Palumbo, E.; Giussani, E.; Monne, I.; Terregino, C.
EPI1998282	Spain	2022-Feb-09	EPI_ISL_11259301	A/turkey/Spain/540-26_22VIR2142-28/2022	Laboratorio Central de Veterinaria	Istituto Zooprofilattico Sperimentale delle Venezie	Ruano, M.J.; Rocha, A.; Sanchez, A.; Aguero, M; Barbierato, G.; Zecchin, B.; Fusaro, A.; Schivo, A.; Salviato, A.; Palumbo, E.; Giussani, E.; Monne, I.; Terregino, C.
EPI1998274	Spain	2022-Feb-08	EPI_ISL_11259300	A/turkey/Spain/490-24_22VIR2142-27/2022	Laboratorio Central de Veterinaria	Istituto Zooprofilattico Sperimentale delle Venezie	Ruano, M.J.; Rocha, A.; Sanchez, A.; Aguero, M; Barbierato, G.; Zecchin, B.; Fusaro, A.; Schivo, A.; Salviato, A.; Palumbo, E.; Giussani, E.; Monne, I.; Terregino, C.
EPI1998266	Spain	2022-Feb-08	EPI_ISL_11259299	A/turkey/Spain/490-22_22VIR2142-26/2022	Laboratorio Central de Veterinaria	Istituto Zooprofilattico Sperimentale delle Venezie	Ruano, M.J.; Rocha, A.; Sanchez, A.; Aguero, M; Barbierato, G.; Zecchin, B.; Fusaro, A.; Schivo, A.; Salviato, A.; Palumbo, E.; Giussani, E.; Monne, I.; Terregino, C.

Segment ID	Country	Collection date	Isolate-ID	Isolate name	Originating Lab	Submitting Lab	Authors
EPI1998258	Spain	2022-Feb-08	EPI_ISL_11259298	A/turkey/Spain/489-21_22VIR2142-25/2022	Laboratorio Central de Veterinaria	Istituto Zooprofilattico Sperimentale delle Venezie	Ruano, M.J.; Rocha, A.; Sanchez, A.; Aguero, M; Barbierato, G.; Zecchin, B.; Fusaro, A.; Schivo, A.; Salviato, A.; Palumbo, E.; Giussani, E.; Monne, I.; Terregino, C.
EPI1998250	Spain	2022-Feb-06	EPI_ISL_11259297	A/turkey/Spain/455-96_22VIR2142-24/2022	Laboratorio Central de Veterinaria	Istituto Zooprofilattico Sperimentale delle Venezie	Ruano, M.J.; Rocha, A.; Sanchez, A.; Aguero, M; Barbierato, G.; Zecchin, B.; Fusaro, A.; Schivo, A.; Salviato, A.; Palumbo, E.; Giussani, E.; Monne, I.; Terregino, C.
EPI1998242	Spain	2022-Feb-06	EPI_ISL_11259296	A/turkey/Spain/455-83_22VIR2142-23/2022	Laboratorio Central de Veterinaria	Istituto Zooprofilattico Sperimentale delle Venezie	Ruano, M.J.; Rocha, A.; Sanchez, A.; Aguero, M; Barbierato, G.; Zecchin, B.; Fusaro, A.; Schivo, A.; Salviato, A.; Palumbo, E.; Giussani, E.; Monne, I.; Terregino, C.
EPI1998202	Spain	2022-Jan-15	EPI_ISL_11259291	A/turkey/Spain/140-38_22VIR2142-19/2022	Laboratorio Central de Veterinaria	Istituto Zooprofilattico Sperimentale delle Venezie	Ruano, M.J.; Rocha, A.; Sanchez, A.; Aguero, M; Barbierato, G.; Zecchin, B.; Fusaro, A.; Schivo, A.; Salviato, A.; Palumbo, E.; Giussani, E.; Monne, I.; Terregino, C.
EPI1998025	Ireland	2021-Nov-19	EPI_ISL_11259269	A/turkey/Ireland/033674_22VIR1325-19/2021	Central Veterinary Research Laboratory	Istituto Zooprofilattico Sperimentale delle Venezie	Byrne, C.; Garcia, K.; Cuartero, L.G.; Barbierato, G.; Zecchin, B.; Fusaro, A.; Schivo, A.; Salviato, A.; Palumbo, E.; Giussani, E.; Monne, I.; Terregino, C.
EPI1998009	Ireland	2021-Dec-06	EPI_ISL_11259267	A/turkey/Ireland/035425_22VIR1325-17/2021	Central Veterinary Research Laboratory	Istituto Zooprofilattico Sperimentale delle Venezie	Byrne, C.; Garcia, K.; Cuartero, L.G.; Barbierato, G.; Zecchin, B.; Fusaro, A.; Schivo, A.; Salviato, A.; Palumbo, E.; Giussani, E.; Monne, I.; Terregino, C.
EPI1995156	Bulgaria	2021-Nov-30	EPI_ISL_11007539	A/turkey/Bulgaria/755-1_22VIR778-4/2021	NDRVMI (National Diagnostic and Research Veterinary Medical Institute)	Istituto Zooprofilattico Sperimentale delle Venezie	Goujgoulova, G.; Slavcheva, I.; Oreshkova, L.; Barbierato, G.; Zecchin, B.; Fusaro, A.; Schivo, A.; Salviato, A.; Palumbo, E.; Giussani, E.; Monne, I.; Terregino, C.
EPI1945187	Germany	2021-Oct-20	EPI_ISL_7753290	A/turkey/Germany-MV/AI06035/2021	Landesamt für Landwirtschaft, Lebensmittelsicherheit und Fischerei (LALLF)	Friedrich-Loeffler-Institut	NA
EPI1943844	Germany	2021-May-03	EPI_ISL_5146481	A/turkey/Germany-NI/AI04425/2021	Lebensmittel- und Veterinärinstitut Oldenburg - Standort Veterinärinstitut	Friedrich-Loeffler-Institut	NA
EPI1943845	Germany	2021-Apr-27	EPI_ISL_5146292	A/turkey/Germany-NI/AI04373/2021	Lebensmittel- und Veterinärinstitut Oldenburg - Standort Veterinärinstitut	Friedrich-Loeffler-Institut	NA
EPI1943857	Germany	2021-May-05	EPI_ISL_5145223	A/turkey/Germany-NI/AI04455/2021	Lebensmittel- und Veterinärinstitut Oldenburg - Standort Veterinärinstitut	Friedrich-Loeffler-Institut	NA

Segment ID	Country	Collection date	Isolate-ID	Isolate name	Originating Lab	Submitting Lab	Authors
EPI1998430	Ireland	2021-Dec-22	EPI_ISL_11260218	A/mute_swan/Ireland/037311_22VIR1325-13/2021	Central Veterinary Research Laboratory	Istituto Zooprofilattico Sperimentale delle Venezie	Byrne, C.; Garcia, K.; Cuartero, L.G.; Barbierato, G.; Zecchin, B.; Fusaro, A.; Schivo, A.; Salviato, A.; Palumbo, E.; Giussani, E.; Monne, I.; Terregino, C.
EPI1998410	Spain	2022-Jan-17	EPI_ISL_11259317	A/stork/Spain/234-2_22VIR2142-7/2022	Laboratorio Central de Veterinaria	Istituto Zooprofilattico Sperimentale delle Venezie	Ruano, M.J.; Rocha, A.; Sanchez, A.; Aguero, M; Barbierato, G.; Zecchin, B.; Fusaro, A.; Schivo, A.; Salviato, A.; Palumbo, E.; Giussani, E.; Monne, I.; Terregino, C.
EPI1998378	Spain	2022-Jan-11	EPI_ISL_11259313	A/gray_heron/Spain/88-2_22VIR2142-3/2022	Laboratorio Central de Veterinaria	Istituto Zooprofilattico Sperimentale delle Venezie	Ruano, M.J.; Rocha, A.; Sanchez, A.; Aguero, M; Barbierato, G.; Zecchin, B.; Fusaro, A.; Schivo, A.; Salviato, A.; Palumbo, E.; Giussani, E.; Monne, I.; Terregino, C.
EPI1998194	Spain	2022-Feb-22	EPI_ISL_11259290	A/stork/Spain/729-1_22VIR2142-18/2022	Laboratorio Central de Veterinaria	Istituto Zooprofilattico Sperimentale delle Venezie	Ruano, M.J.; Rocha, A.; Sanchez, A.; Aguero, M; Barbierato, G.; Zecchin, B.; Fusaro, A.; Schivo, A.; Salviato, A.; Palumbo, E.; Giussani, E.; Monne, I.; Terregino, C.
EPI1998186	Spain	2022-Feb-10	EPI_ISL_11259289	A/gray_heron/Spain/602-1_22VIR2142-17/2022	Laboratorio Central de Veterinaria	Istituto Zooprofilattico Sperimentale delle Venezie	Ruano, M.J.; Rocha, A.; Sanchez, A.; Aguero, M; Barbierato, G.; Zecchin, B.; Fusaro, A.; Schivo, A.; Salviato, A.; Palumbo, E.; Giussani, E.; Monne, I.; Terregino, C.
EPI1998178	Spain	2022-Feb-14	EPI_ISL_11259288	A/common_crane/Spain/597-2_22VIR2142-15/2022	Laboratorio Central de Veterinaria	Istituto Zooprofilattico Sperimentale delle Venezie	Ruano, M.J.; Rocha, A.; Sanchez, A.; Aguero, M; Barbierato, G.; Zecchin, B.; Fusaro, A.; Schivo, A.; Salviato, A.; Palumbo, E.; Giussani, E.; Monne, I.; Terregino, C.
EPI1998170	Spain	2022-Feb-03	EPI_ISL_11259287	A/stork/Spain/538-2_22VIR2142-14/2022	Laboratorio Central de Veterinaria	Istituto Zooprofilattico Sperimentale delle Venezie	Ruano, M.J.; Rocha, A.; Sanchez, A.; Aguero, M; Barbierato, G.; Zecchin, B.; Fusaro, A.; Schivo, A.; Salviato, A.; Palumbo, E.; Giussani, E.; Monne, I.; Terregino, C.
EPI1998154	Spain	2022-Feb-03	EPI_ISL_11259285	A/stork/Spain/442-8_22VIR2142-12/2022	Laboratorio Central de Veterinaria	Istituto Zooprofilattico Sperimentale delle Venezie	Ruano, M.J.; Rocha, A.; Sanchez, A.; Aguero, M; Barbierato, G.; Zecchin, B.; Fusaro, A.; Schivo, A.; Salviato, A.; Palumbo, E.; Giussani, E.; Monne, I.; Terregino, C.
EPI1998130	Ireland	2021-Nov-16	EPI_ISL_11259282	A/brent_goose/Ireland/033257_22VIR1325-9/2021	Central Veterinary Research Laboratory	Istituto Zooprofilattico Sperimentale delle Venezie	Byrne, C.; Garcia, K.; Cuartero, L.G.; Barbierato, G.; Zecchin, B.; Fusaro, A.; Schivo, A.; Salviato, A.; Palumbo, E.; Giussani, E.; Monne, I.; Terregino, C.
EPI1998122	Ireland	2021-Nov-15	EPI_ISL_11259281	A/white-fronted_goose/Ireland/033181_22VIR1325-8/2021	Central Veterinary Research Laboratory	Istituto Zooprofilattico Sperimentale delle Venezie	Byrne, C.; Garcia, K.; Cuartero, L.G.; Barbierato, G.; Zecchin, B.; Fusaro, A.; Schivo, A.; Salviato, A.; Palumbo, E.; Giussani, E.; Monne, I.; Terregino, C.
EPI1998114	Ireland	2021-Nov-15	EPI_ISL_11259280	A/greylag_goose/Ireland/033062_22VIR1325-7/2021	Central Veterinary Research Laboratory	Istituto Zooprofilattico Sperimentale delle Venezie	Byrne, C.; Garcia, K.; Cuartero, L.G.; Barbierato, G.; Zecchin, B.; Fusaro, A.; Schivo, A.; Salviato, A.; Palumbo, E.; Giussani, E.; Monne, I.; Terregino, C.

Segment ID	Country	Collection date	Isolate-ID	Isolate name	Originating Lab	Submitting Lab	Authors
EPI1998106	Ireland	2021-Nov-15	EPI_ISL_11259279	A/greylag_goose/Ireland/032969_22VIR1325-6/2021	Central Veterinary Research Laboratory	Istituto Zooprofilattico Sperimentale delle Venezie	Byrne, C.; Garcia, K.; Cuartero, L.G.; Barbierato, G.; Zecchin, B.; Fusaro, A.; Schivo, A.; Salviato, A.; Palumbo, E.; Giussani, E.; Monne, I.; Terregino, C.
EPI1998098	Ireland	2021-Nov-12	EPI_ISL_11259278	A/magpie/Ireland/032958_22VIR1325-5/2021	Central Veterinary Research Laboratory	Istituto Zooprofilattico Sperimentale delle Venezie	Byrne, C.; Garcia, K.; Cuartero, L.G.; Barbierato, G.; Zecchin, B.; Fusaro, A.; Schivo, A.; Salviato, A.; Palumbo, E.; Giussani, E.; Monne, I.; Terregino, C.
EPI1998090	Ireland	2021-Nov-12	EPI_ISL_11259277	A/whooper_swan/Ireland/032960_22VIR1325-4/2021	Central Veterinary Research Laboratory	Istituto Zooprofilattico Sperimentale delle Venezie	Byrne, C.; Garcia, K.; Cuartero, L.G.; Barbierato, G.; Zecchin, B.; Fusaro, A.; Schivo, A.; Salviato, A.; Palumbo, E.; Giussani, E.; Monne, I.; Terregino, C.
EPI1998082	Ireland	2021-Nov-10	EPI_ISL_11259276	A/peregrine_falcon/Ireland/032476_22VIR1325-3/2021	Central Veterinary Research Laboratory	Istituto Zooprofilattico Sperimentale delle Venezie	Byrne, C.; Garcia, K.; Cuartero, L.G.; Barbierato, G.; Zecchin, B.; Fusaro, A.; Schivo, A.; Salviato, A.; Palumbo, E.; Giussani, E.; Monne, I.; Terregino, C.
EPI1998074	Ireland	2021-Nov-09	EPI_ISL_11259275	A/whooper_swan/Ireland/032444_22VIR1325-2/2021	Central Veterinary Research Laboratory	Istituto Zooprofilattico Sperimentale delle Venezie	Byrne, C.; Garcia, K.; Cuartero, L.G.; Barbierato, G.; Zecchin, B.; Fusaro, A.; Schivo, A.; Salviato, A.; Palumbo, E.; Giussani, E.; Monne, I.; Terregino, C.
EPI1998066	Ireland	2021-Nov-03	EPI_ISL_11259274	A/white-tailed_eagle/Ireland/032034_22VIR1325-23/2021	Central Veterinary Research Laboratory	Istituto Zooprofilattico Sperimentale delle Venezie	Byrne, C.; Garcia, K.; Cuartero, L.G.; Barbierato, G.; Zecchin, B.; Fusaro, A.; Schivo, A.; Salviato, A.; Palumbo, E.; Giussani, E.; Monne, I.; Terregino, C.
EPI1998050	Ireland	2021-Nov-29	EPI_ISL_11259272	A/layer/Ireland/034424_22VIR1325-21/2021	Central Veterinary Research Laboratory	Istituto Zooprofilattico Sperimentale delle Venezie	Byrne, C.; Garcia, K.; Cuartero, L.G.; Barbierato, G.; Zecchin, B.; Fusaro, A.; Schivo, A.; Salviato, A.; Palumbo, E.; Giussani, E.; Monne, I.; Terregino, C.
EPI1998033	Ireland	2021-Nov-08	EPI_ISL_11259270	A/mute_swan/Ireland/032363_22VIR1325-1/2021	Central Veterinary Research Laboratory	Istituto Zooprofilattico Sperimentale delle Venezie	Byrne, C.; Garcia, K.; Cuartero, L.G.; Barbierato, G.; Zecchin, B.; Fusaro, A.; Schivo, A.; Salviato, A.; Palumbo, E.; Giussani, E.; Monne, I.; Terregino, C.
EPI1998001	Ireland	2021-Nov-19	EPI_ISL_11259266	A/mute_swan/Ireland/033945_22VIR1325-16/2021	Central Veterinary Research Laboratory	Istituto Zooprofilattico Sperimentale delle Venezie	Byrne, C.; Garcia, K.; Cuartero, L.G.; Barbierato, G.; Zecchin, B.; Fusaro, A.; Schivo, A.; Salviato, A.; Palumbo, E.; Giussani, E.; Monne, I.; Terregino, C.
EPI1997993	Ireland	2022-Jan-05	EPI_ISL_11259265	A/peregrine_falcon/Ireland/000191_22VIR1325-15/2022	Central Veterinary Research Laboratory	Istituto Zooprofilattico Sperimentale delle Venezie	Byrne, C.; Garcia, K.; Cuartero, L.G.; Barbierato, G.; Zecchin, B.; Fusaro, A.; Schivo, A.; Salviato, A.; Palumbo, E.; Giussani, E.; Monne, I.; Terregino, C.
EPI1997985	Ireland	2021-Nov-15	EPI_ISL_11259264	A/mute_swan/Ireland/033169_22VIR1325-14/2021	Central Veterinary Research Laboratory	Istituto Zooprofilattico Sperimentale delle Venezie	Byrne, C.; Garcia, K.; Cuartero, L.G.; Barbierato, G.; Zecchin, B.; Fusaro, A.; Schivo, A.; Salviato, A.; Palumbo, E.; Giussani, E.; Monne, I.; Terregino, C.

Segment ID	Country	Collection date	Isolate-ID	Isolate name	Originating Lab	Submitting Lab	Authors
EPI1997977	Ireland	2022-Jan-07	EPI_ISL_11259263	A/buzzard/Ireland/000656_22VIR1325-12/2022	Central Veterinary Research Laboratory	Istituto Zooprofilattico Sperimentale delle Venezie	Byrne, C.; Garcia, K.; Cuartero, L.G.; Barbierato, G.; Zecchin, B.; Fusaro, A.; Schivo, A.; Salviato, A.; Palumbo, E.; Giussani, E.; Monne, I.; Terregino, C.
EPI1997969	Ireland	2021-Dec-08	EPI_ISL_11259262	A/crow/Ireland/035624_22VIR1325-11/2021	Central Veterinary Research Laboratory	Istituto Zooprofilattico Sperimentale delle Venezie	Byrne, C.; Garcia, K.; Cuartero, L.G.; Barbierato, G.; Zecchin, B.; Fusaro, A.; Schivo, A.; Salviato, A.; Palumbo, E.; Giussani, E.; Monne, I.; Terregino, C.
EPI1997961	Ireland	2021-Nov-17	EPI_ISL_11259261	A/herring_gull/Ireland/033533_22VIR1325-10/2021	Central Veterinary Research Laboratory	Istituto Zooprofilattico Sperimentale delle Venezie	Byrne, C.; Garcia, K.; Cuartero, L.G.; Barbierato, G.; Zecchin, B.; Fusaro, A.; Schivo, A.; Salviato, A.; Palumbo, E.; Giussani, E.; Monne, I.; Terregino, C.
EPI1995236	Moldova, Republic of	2022-Jan-03	EPI_ISL_11007721	A/laying_hen/Moldova/68-2_22VIR638-2/2022	Republican Center of Veterinary Diagnostics	Istituto Zooprofilattico Sperimentale delle Venezie	Arseniev, S.; Groza, O.; Barbierato, G.; Zecchin, B.; Fusaro, A.; Schivo, A.; Salviato, A.; Palumbo, E.; Giussani, E.; Monne, I.; Terregino, C.
EPI1995172	Bulgaria	2021-Dec-02	EPI_ISL_11007541	A/hen/Bulgaria/757-6_22VIR778-7/2021	NDRVMI (National Diagnostic and Research Veterinary Medical Institute)	Istituto Zooprofilattico Sperimentale delle Venezie	Goujgoulova, G.; Slavcheva, I.; Oreshkova, L.; Barbierato, G.; Zecchin, B.; Fusaro, A.; Schivo, A.; Salviato, A.; Palumbo, E.; Giussani, E.; Monne, I.; Terregino, C.
EPI1995148	Bulgaria	2021-Nov-15	EPI_ISL_11007538	A/hen/Bulgaria/722-1_22VIR778-1/2021	NDRVMI (National Diagnostic and Research Veterinary Medical Institute)	Istituto Zooprofilattico Sperimentale delle Venezie	Goujgoulova, G.; Slavcheva, I.; Oreshkova, L.; Barbierato, G.; Zecchin, B.; Fusaro, A.; Schivo, A.; Salviato, A.; Palumbo, E.; Giussani, E.; Monne, I.; Terregino, C.
EPI1995140	Bulgaria	2021-Dec-29	EPI_ISL_11007537	A/hen/Bulgaria/854-1_22VIR778-10/2021	NDRVMI (National Diagnostic and Research Veterinary Medical Institute)	Istituto Zooprofilattico Sperimentale delle Venezie	Goujgoulova, G.; Slavcheva, I.; Oreshkova, L.; Barbierato, G.; Zecchin, B.; Fusaro, A.; Schivo, A.; Salviato, A.; Palumbo, E.; Giussani, E.; Monne, I.; Terregino, C.
EPI1995116	Slovenia	2021-Dec-30	EPI_ISL_11007534	A/seagull/Slovenia/2075_22VIR777-7/2021	University of Ljubljana	Istituto Zooprofilattico Sperimentale delle Venezie	Slavec, B.; Ra?nik, J.; Krape?, U.; ?labravec, Z.; A?ko, J.; Cociancich, V.; Paller, T.; Vidrih, ?.; Rojs, O.Z.; Arseniev, S.; Groza, O.; Barbierato, G.; Zecchin, B.; Fusaro, A.; Schivo, A.; Salviato, A.; Palumbo, E.; Giussani, E.; Monne, I.; Terregino, C.
EPI1995068	Slovenia	2021-Dec-26	EPI_ISL_11007528	A/rooster/Slovenia/2039_22VIR777-1/2021	University of Ljubljana	Istituto Zooprofilattico Sperimentale delle Venezie	Slavec, B.; Ra?nik, J.; Krape?, U.; ?labravec, Z.; A?ko, J.; Cociancich, V.; Paller, T.; Vidrih, ?.; Rojs, O.Z.; Arseniev, S.; Groza, O.; Barbierato, G.; Zecchin, B.; Fusaro, A.; Schivo, A.; Salviato, A.; Palumbo, E.; Giussani, E.; Monne, I.; Terregino, C.
EPI1995060	Moldova, Republic of	2022-Jan-03	EPI_ISL_11007527	A/laying_hen/Moldova/68-1_22VIR638-1/2022	Republican Center of Veterinary Diagnostics	Istituto Zooprofilattico Sperimentale delle Venezie	Arseniev, S.; Groza, O.; Barbierato, G.; Zecchin, B.; Fusaro, A.; Schivo, A.; Salviato, A.; Palumbo, E.; Giussani, E.; Monne, I.; Terregino, C.
EPI1949882	France	2021-Dec-03	EPI_ISL_8377254	A/egret/France/21P013418/2021	Anses (Ploufragan-Plouzané)	ANSES Agence Nationale De Securite Sanitaire De L'alimentation	NA
EPI1943854	Germany	2021-Jul-17	EPI_ISL_5145744	A/oystercatcher/Germany-NI/AI05047/2021	Lebensmittel- und Veterinärinstitut	Friedrich-Loeffler-Institut	NA

Segment ID	Country	Collection date	Isolate-ID	Isolate name	Originating Lab	Submitting Lab	Authors
					Oldenburg - Standort Veterinärinstitut		
EPI1998138	Ireland	2022-Feb-14	EPI_ISL_11259283	A/fox/Ireland/3866_22VIR2064-1/2022	Central Veterinary Research Laboratory	Istituto Zooprofilattico Sperimentale delle Venezie	Byrne, C.; Garcia, K.; Cuartero, L.G.; Barbierato, G.; Zecchin, B.; Fusaro, A.; Schivo, A.; Salviato, A.; Palumbo, E.; Giussani, E.; Monne, I.; Terregino, C.
EPI1945585	Estonia	2021-Nov-08	EPI_ISL_7778880	A/Red_fox/Estonia/TA2126820_21VIR10433-13/2021	Estonian Veterinary and Food Laboratory	Istituto Zooprofilattico Sperimentale Delle Venezie	Nurmoja, I.; Vilem, A.; Juurik, T.; Zecchin, B.; Fusaro, A.; Schivo, A.; Salviato, A.; Palumbo, E.; Milani, A.; Giussani, E.; Pastori, A.; Monne, I.; Terregino, C.
EPI1958565	United Kingdom	2021-Dec-26	EPI_ISL_8799552	A/England/215201407/2021	UK Health Security Agency - Colindale	UK Health Security Agency (UKHSA)	UKHSA, Respiratory Virus Unit
EPI1995045	Netherlands	2022-Jan-29	EPI_ISL_10993196	A/Sanderling/Netherlands/1/2022	Erasmus Medical Center	Erasmus Medical Center	NA
EPI1995037	Netherlands	2021-Nov-29	EPI_ISL_10993166	A/Mallard/Netherlands/8/2021	Erasmus Medical Center	Erasmus Medical Center	NA
EPI1995029	Netherlands	2021-Nov-29	EPI_ISL_10993134	A/Mallard/Netherlands/7/2021	Erasmus Medical Center	Erasmus Medical Center	NA
EPI1995021	Netherlands	2021-Nov-29	EPI_ISL_10993105	A/Mallard/Netherlands/6/2021	Erasmus Medical Center	Erasmus Medical Center	NA
EPI1995013	Netherlands	2021-Nov-29	EPI_ISL_10993076	A/Mallard/Netherlands/5/2021	Erasmus Medical Center	Erasmus Medical Center	NA
EPI1995005	Netherlands	2021-Nov-29	EPI_ISL_10993075	A/Mallard/Netherlands/4/2021	Erasmus Medical Center	Erasmus Medical Center	NA
EPI1994997	Netherlands	2022-Feb-14	EPI_ISL_10993074	A/Mallard/Netherlands/2/2022	Erasmus Medical Center	Erasmus Medical Center	NA
EPI1994989	Netherlands	2022-Feb-12	EPI_ISL_10992813	A/Mallard/Netherlands/1/2022	Erasmus Medical Center	Erasmus Medical Center	NA
EPI1966970	Netherlands	2022-Jan-11	EPI_ISL_9261743	A/Oystercatcher/Netherlands/1/2022	Erasmus Medical Center	Erasmus Medical Center	NA
EPI1963368	Netherlands	2021-Dec-27	EPI_ISL_9012580	A/Mallard/Netherlands/15/2021	Erasmus Medical Center	Erasmus Medical Center	NA
EPI1963360	Netherlands	2021-Dec-27	EPI_ISL_9012578	A/Mallard/Netherlands/14/2021	Erasmus Medical Center	Erasmus Medical Center	NA
EPI1963352	Netherlands	2021-Dec-27	EPI_ISL_9012577	A/Mallard/Netherlands/13/2021	Erasmus Medical Center	Erasmus Medical Center	NA
EPI1945291	Germany	2021-Oct-26	EPI_ISL_7753443	A/buzzard/Germany-SH/AI06210/2021	Landeslabor Schleswig-Holstein	Friedrich-Loeffler-Institut	NA
EPI1877873	Poland	2021-Apr-20	EPI_ISL_2681045	A/white_stork/Poland/MB391/2021	National Veterinary Research Institut Poland, PIWet-PIB	National Veterinary Research Institut Poland, PIWet-PIB	Edyta, Swieton; Kamila, Dziadek; Krzysztof, Smietanka
EPI2014961	Poland	2022-Jan-23	EPI_ISL_11922812	A/chicken/Poland/H071_22VIR2515-6/2022	National Veterinary Research Institute	Istituto Zooprofilattico	Swieton, E.; Smietanka, K.; Barbierato, G.; Zecchin, B.; Fusaro, A.; Schivo, A.; Salviato, A.; Palumbo, E.; Giussani, E.; Monne, I.; Terregino, C.

Segment ID	Country	Collection date	Isolate-ID	Isolate name	Originating Lab	Submitting Lab	Authors
						Sperimentale delle Venezie	
EPI2014937	Poland	2022-Feb-18	EPI_ISL_11922809	A/chicken/Poland/H157_22VIR2515-3/2022	National Veterinary Research Institute	Istituto Zooprofilattico Sperimentale delle Venezie	Swieton, E.; Smietanka, K.; Barbierato, G.; Zecchin, B.; Fusaro, A.; Schivo, A.; Salviato, A.; Palumbo, E.; Giussani, E.; Monne, I.; Terregino, C.
EPI2009951	Germany	2021-Nov-02	EPI_ISL_11725988	A/chicken/Germany-BB/AI06242/2021	Landeslabor Berlin-Brandenburg	Friedrich-Loeffler-Institut	NA
EPI2009943	Germany	2021-Oct-29	EPI_ISL_11725962	A/chicken/Germany-BB/AI06219/2021	Landeslabor Berlin-Brandenburg	Friedrich-Loeffler-Institut	NA
EPI1998370	Spain	2022-Feb-16	EPI_ISL_11259312	A/chicken/Spain/649-6_22VIR2142-38/2022	Laboratorio Central de Veterinaria	Istituto Zooprofilattico Sperimentale delle Venezie	Ruano, M.J.; Rocha, A.; Sanchez, A.; Aguero, M; Barbierato, G.; Zecchin, B.; Fusaro, A.; Schivo, A.; Salviato, A.; Palumbo, E.; Giussani, E.; Monne, I.; Terregino, C.
EPI1998346	Spain	2022-Feb-16	EPI_ISL_11259309	A/chicken/Spain/644-8_22VIR2142-35/2022	Laboratorio Central de Veterinaria	Istituto Zooprofilattico Sperimentale delle Venezie	Ruano, M.J.; Rocha, A.; Sanchez, A.; Aguero, M; Barbierato, G.; Zecchin, B.; Fusaro, A.; Schivo, A.; Salviato, A.; Palumbo, E.; Giussani, E.; Monne, I.; Terregino, C.
EPI1998338	Spain	2022-Feb-14	EPI_ISL_11259308	A/chicken/Spain/622-8_22VIR2142-34/2022	Laboratorio Central de Veterinaria	Istituto Zooprofilattico Sperimentale delle Venezie	Ruano, M.J.; Rocha, A.; Sanchez, A.; Aguero, M; Barbierato, G.; Zecchin, B.; Fusaro, A.; Schivo, A.; Salviato, A.; Palumbo, E.; Giussani, E.; Monne, I.; Terregino, C.
EPI1998330	Spain	2022-Feb-14	EPI_ISL_11259307	A/chicken/Spain/587-1_22VIR2142-33/2022	Laboratorio Central de Veterinaria	Istituto Zooprofilattico Sperimentale delle Venezie	Ruano, M.J.; Rocha, A.; Sanchez, A.; Aguero, M; Barbierato, G.; Zecchin, B.; Fusaro, A.; Schivo, A.; Salviato, A.; Palumbo, E.; Giussani, E.; Monne, I.; Terregino, C.
EPI1998314	Spain	2022-Feb-11	EPI_ISL_11259305	A/chicken/Spain/564-11_22VIR2142-31/2022	Laboratorio Central de Veterinaria	Istituto Zooprofilattico Sperimentale delle Venezie	Ruano, M.J.; Rocha, A.; Sanchez, A.; Aguero, M; Barbierato, G.; Zecchin, B.; Fusaro, A.; Schivo, A.; Salviato, A.; Palumbo, E.; Giussani, E.; Monne, I.; Terregino, C.
EPI1998306	Spain	2022-Feb-11	EPI_ISL_11259304	A/chicken/Spain/564-4_22VIR2142-30/2022	Laboratorio Central de Veterinaria	Istituto Zooprofilattico Sperimentale delle Venezie	Ruano, M.J.; Rocha, A.; Sanchez, A.; Aguero, M; Barbierato, G.; Zecchin, B.; Fusaro, A.; Schivo, A.; Salviato, A.; Palumbo, E.; Giussani, E.; Monne, I.; Terregino, C.
EPI1998290	Spain	2022-Feb-11	EPI_ISL_11259302	A/chicken/Spain/562-1_22VIR2142-29/2022	Laboratorio Central de Veterinaria	Istituto Zooprofilattico Sperimentale delle Venezie	Ruano, M.J.; Rocha, A.; Sanchez, A.; Aguero, M; Barbierato, G.; Zecchin, B.; Fusaro, A.; Schivo, A.; Salviato, A.; Palumbo, E.; Giussani, E.; Monne, I.; Terregino, C.
EPI1998234	Spain	2022-Feb-07	EPI_ISL_11259295	A/chicken/Spain/452-17_22VIR2142-22/2022	Laboratorio Central de Veterinaria	Istituto Zooprofilattico Sperimentale delle Venezie	Ruano, M.J.; Rocha, A.; Sanchez, A.; Aguero, M; Barbierato, G.; Zecchin, B.; Fusaro, A.; Schivo, A.; Salviato, A.; Palumbo, E.; Giussani, E.; Monne, I.; Terregino, C.
EPI1998226	Spain	2022-Feb-07	EPI_ISL_11259294	A/chicken/Spain/452-1_22VIR2142-21/2022	Laboratorio Central de Veterinaria	Istituto Zooprofilattico Sperimentale delle Venezie	Ruano, M.J.; Rocha, A.; Sanchez, A.; Aguero, M; Barbierato, G.; Zecchin, B.; Fusaro, A.; Schivo, A.; Salviato, A.; Palumbo, E.; Giussani, E.; Monne, I.; Terregino, C.

Segment ID	Country	Collection date	Isolate-ID	Isolate name	Originating Lab	Submitting Lab	Authors
EPI1998218	Spain	2022-Jan-31	EPI_ISL_11259293	A/chicken/Spain/340-37_22VIR2142-20/2022	Laboratorio Central de Veterinaria	Istituto Zooprofilattico Sperimentale delle Venezie	Ruano, M.J.; Rocha, A.; Sanchez, A.; Aguero, M; Barbierato, G.; Zecchin, B.; Fusaro, A.; Schivo, A.; Salviato, A.; Palumbo, E.; Giussani, E.; Monne, I.; Terregino, C.
EPI1998042	Ireland	2021-Nov-22	EPI_ISL_11259271	A/broiler/Ireland/033734_22VIR1325-20/2021	Central Veterinary Research Laboratory	Istituto Zooprofilattico Sperimentale delle Venezie	Byrne, C.; Garcia, K.; Cuartero, L.G.; Barbierato, G.; Zecchin, B.; Fusaro, A.; Schivo, A.; Salviato, A.; Palumbo, E.; Giussani, E.; Monne, I.; Terregino, C.
EPI1949890	France	2021-Nov-25	EPI_ISL_8377417	A/chicken/France/21P013076/2021	Anses (Ploufragan-Plouzané)	ANSES Agence Nationale De Securite Sanitaire De L'alimentation	NA
EPI1944199	Germany	2021-Feb-23	EPI_ISL_5099451	A/chicken/Germany-NI/AI01599/2021	Lebensmittel- und Veterinärinstitut Oldenburg - Standort Veterinärinstitut	Friedrich-Loeffler-Institut	NA
EPI1985904	Netherlands	2021-Oct-25	EPI_ISL_9856775	A/chicken/Netherlands/21037287-006010/2021	Wageningen Bioveterinary Research	Wageningen Bioveterinary Research	Beerens, Nancy; Harders, Frank; Pritz-Verschuren, Sylvia; Roose, Marit; Venema, Sandra; Germeraad, Evelien; Engelsma, Marc; Heutink, Rene; Luca, Bordes
EPI1965237	Netherlands	2022-Jan-22	EPI_ISL_9111105	A/chicken/Netherlands/22001401-001005/2022	Wageningen Bioveterinary Research	Wageningen Bioveterinary Research	Beerens, Nancy; Harders, Frank; Pritz-Verschuren, Sylvia; Roose, Marit; Venema, Sandra; Germeraad, Evelien; Engelsma, Marc; Heutink, Rene
EPI1923252	Netherlands	2021-Oct-22	EPI_ISL_5588106	A/chicken/Netherlands/21037233-001/2021	Wageningen Bioveterinary Research	Wageningen Bioveterinary Research	Beerens, Nancy; Harders, Frank; Pritz-Verschuren, Sylvia; Roose, Marit; Germeraad, Evelien; Engelsma, Marc; Heutink, Rene
EPI1923244	Netherlands	2021-Oct-25	EPI_ISL_5588100	A/chicken/Netherlands/21037287-006010/2021	Wageningen Bioveterinary Research	Wageningen Bioveterinary Research	Beerens, Nancy; Harders, Frank; Pritz-Verschuren, Sylvia; Roose, Marit; Germeraad, Evelien; Engelsma, Marc; Heutink, Rene
EPI1838673	Netherlands	2020-Dec-14	EPI_ISL_711055	A/chicken/Netherlands/20019879-001005/2020	Wageningen Bioveterinary Research	Wageningen Bioveterinary Research	Beerens, Nancy; Harders, Frank; Pritz-Verschuren, Sylvia; Roose, Marit; Germeraad, Evelien; Engelsma, Marc; Bossers, Alex; Heutink, Rene
EPI2014945	Poland	2022-Feb-08	EPI_ISL_11922810	A/duck/Poland/H126_22VIR2515-4/2022	National Veterinary Research Institute	Istituto Zooprofilattico Sperimentale delle Venezie	Swieton, E.; Smietanka, K.; Barbierato, G.; Zecchin, B.; Fusaro, A.; Schivo, A.; Salviato, A.; Palumbo, E.; Giussani, E.; Monne, I.; Terregino, C.
EPI2014929	Poland	2022-Mar-02	EPI_ISL_11922808	A/duck/Poland/H188_22VIR2515-2/2022	National Veterinary Research Institute	Istituto Zooprofilattico Sperimentale delle Venezie	Swieton, E.; Smietanka, K.; Barbierato, G.; Zecchin, B.; Fusaro, A.; Schivo, A.; Salviato, A.; Palumbo, E.; Giussani, E.; Monne, I.; Terregino, C.
EPI1998058	Ireland	2021-Dec-11	EPI_ISL_11259273	A/duck/Ireland/036105_22VIR1325-22/2021	Central Veterinary Research Laboratory	Istituto Zooprofilattico Sperimentale delle Venezie	Byrne, C.; Garcia, K.; Cuartero, L.G.; Barbierato, G.; Zecchin, B.; Fusaro, A.; Schivo, A.; Salviato, A.; Palumbo, E.; Giussani, E.; Monne, I.; Terregino, C.
EPI1998017	Ireland	2021-Dec-17	EPI_ISL_11259268	A/duck/Ireland/036646_22VIR1325-18/2021	Central Veterinary Research Laboratory	Istituto Zooprofilattico	Byrne, C.; Garcia, K.; Cuartero, L.G.; Barbierato, G.; Zecchin, B.; Fusaro, A.; Schivo, A.; Salviato, A.; Palumbo, E.; Giussani, E.; Monne, I.; Terregino, C.

Segment ID	Country	Collection date	Isolate-ID	Isolate name	Originating Lab	Submitting Lab	Authors
						Sperimentale delle Venezie	
EPI1995256	Bulgaria	2021-Dec-16	EPI_ISL_11009382	A/duck/Bulgaria/827-2_22VIR778-8/2021	NDRVMI (National Diagnostic and Research Veterinary Medical Institute)	Istituto Zooprofilattico Sperimentale Delle Venezie	Goujgoulova, G.; Slavcheva, I.; Oreshkova, L.; Barbierato, G.; Zecchin, B.; Fusaro, A.; Schivo, A.; Salviato, A.; Palumbo, E.; Giussani, E.; Monne, I.; Terregino, C.
EPI1995164	Bulgaria	2021-Dec-01	EPI_ISL_11007540	A/duck/Bulgaria/756-4_22VIR778-6/2021	NDRVMI (National Diagnostic and Research Veterinary Medical Institute)	Istituto Zooprofilattico Sperimentale delle Venezie	Goujgoulova, G.; Slavcheva, I.; Oreshkova, L.; Barbierato, G.; Zecchin, B.; Fusaro, A.; Schivo, A.; Salviato, A.; Palumbo, E.; Giussani, E.; Monne, I.; Terregino, C.
EPI1945163	Germany	2021-Oct-17	EPI_ISL_7753230	A/mallard/Germany-NI/AI06010/2021	Lebensmittel- und Veterinärinstitut Oldenburg - Standort Veterinärinstitut	Friedrich-Loeffler-Institut	NA
EPI1963873	United Kingdom	2021-Nov-18	EPI_ISL_9029959	A/domestic_duck/England/058612/2021	Animal and Plant Health Agency (APHA)	Animal and Plant Health Agency (APHA)	NA
EPI1937655	Poland	2021-Nov-07	EPI_ISL_6934175	A/domestic_duck/Poland/H1942-N/2021	National Veterinary Research Institut Poland, PIWet-PIB	National Veterinary Research Institut Poland, PIWet-PIB	E. Swieton, K. Smietanka
EPI1954257	Croatia	2021-Nov-22	EPI_ISL_8568483	A/gadwall/Croatia/108/2021	Croatian Veterinary Institute, Poultry Centre	Croatian Veterinary Institute	Savić, Vladimir
EPI1963889	United Kingdom	2021-Nov-01	EPI_ISL_9029961	A/Canada_goose/England/385250/2021	Animal and Plant Health Agency (APHA)	Animal and Plant Health Agency (APHA)	NA
EPI1949874	France	2021-Dec-15	EPI_ISL_8377056	A/pelican/France/21P013720/2021	Anses (Ploufragan-Plouzané)	ANSES Agence Nationale De Securite Sanitaire De L'alimentation	NA
EPI1945538	Finland	2021-Jun-01	EPI_ISL_7778775	A/barnacle_goose/Finland/6955_21VIR7689-9/2021	Finnish Food Authority	Istituto Zooprofilattico Sperimentale Delle Venezie	Tammiranta, N.; Kantala, T.; Laamanen, I.; Gadd, T.; Zecchin, B.; Fusaro, A.; Schivo, A.; Salviato, A.; Palumbo, E.; Milani, A.; Giussani, E.; Pastori, A.; Monne, I.; Terregino, C.
EPI1945522	Finland	2021-May-01	EPI_ISL_7778773	A/barnacle_goose/Finland/6247_21VIR7689-6/2021	Finnish Food Authority	Istituto Zooprofilattico Sperimentale Delle Venezie	Tammiranta, N.; Kantala, T.; Laamanen, I.; Gadd, T.; Zecchin, B.; Fusaro, A.; Schivo, A.; Salviato, A.; Palumbo, E.; Milani, A.; Giussani, E.; Pastori, A.; Monne, I.; Terregino, C.
EPI1945485	Finland	2021-Aug-01	EPI_ISL_7778768	A/Eurasian_eagle-owl/Finland/10617_21VIR7689-15/2021	Finnish Food Authority	Istituto Zooprofilattico Sperimentale Delle Venezie	Tammiranta, N.; Kantala, T.; Laamanen, I.; Gadd, T.; Zecchin, B.; Fusaro, A.; Schivo, A.; Salviato, A.; Palumbo, E.; Milani, A.; Giussani, E.; Pastori, A.; Monne, I.; Terregino, C.
EPI1945469	Finland	2021-Aug-01	EPI_ISL_7778766	A/European_herring_gull/Finland/9722_21VIR7689-13/2021	Finnish Food Authority	Istituto Zooprofilattico	Tammiranta, N.; Kantala, T.; Laamanen, I.; Gadd, T.; Zecchin, B.; Fusaro, A.; Schivo, A.; Salviato, A.; Palumbo, E.; Milani, A.; Giussani, E.; Pastori, A.; Monne, I.; Terregino, C.

Segment ID	Country	Collection date	Isolate-ID	Isolate name	Originating Lab	Submitting Lab	Authors
						Sperimentale Delle Venezie	
EPI1945461	Finland	2021-Jul-01	EPI_ISL_7778765	A/golden_eagle/Finland/9378_21VIR7689-12/2021	Finnish Food Authority	Istituto Zooprofilattico Sperimentale Delle Venezie	Tammiranta, N.; Kantala, T.; Laamanen, I.; Gadd, T.; Zecchin, B.; Fusaro, A.; Schivo, A.; Salviato, A.; Palumbo, E.; Milani, A.; Giussani, E.; Pastori, A.; Monne, I.; Terregino, C.
EPI1945445	Finland	2021-Jun-01	EPI_ISL_7778763	A/white-tailed_eagle/Finland/6984_21VIR7689-10/2021	Finnish Food Authority	Istituto Zooprofilattico Sperimentale Delle Venezie	Tammiranta, N.; Kantala, T.; Laamanen, I.; Gadd, T.; Zecchin, B.; Fusaro, A.; Schivo, A.; Salviato, A.; Palumbo, E.; Milani, A.; Giussani, E.; Pastori, A.; Monne, I.; Terregino, C.
EPI1945389	Estonia	2021-Jun-02	EPI_ISL_7778756	A/gull/Estonia/TA2113284-4_21VIR7512-8/2021	Estonian Veterinary and Food Laboratory	Istituto Zooprofilattico Sperimentale Delle Venezie	Nurmoja, I.; Vilem, A.; Juurik, T.; Zecchin, B.; Fusaro, A.; Schivo, A.; Salviato, A.; Palumbo, E.; Milani, A.; Giussani, E.; Pastori, A.; Monne, I.; Terregino, C.
EPI1945381	Estonia	2021-May-16	EPI_ISL_7778755	A/white-tailed_eagle/Estonia/TA2111864-2_21VIR7512-6/2021	Estonian Veterinary and Food Laboratory	Istituto Zooprofilattico Sperimentale Delle Venezie	Nurmoja, I.; Vilem, A.; Juurik, T.; Zecchin, B.; Fusaro, A.; Schivo, A.; Salviato, A.; Palumbo, E.; Milani, A.; Giussani, E.; Pastori, A.; Monne, I.; Terregino, C.
EPI1945373	Estonia	2021-Oct-12	EPI_ISL_7778754	A/Withe-tiled_eagle/Estonia/TA2124126-1_21VIR10433-11/2021	Estonian Veterinary and Food Laboratory	Istituto Zooprofilattico Sperimentale Delle Venezie	Nurmoja, I.; Vilem, A.; Juurik, T.; Zecchin, B.; Fusaro, A.; Schivo, A.; Salviato, A.; Palumbo, E.; Milani, A.; Giussani, E.; Pastori, A.; Monne, I.; Terregino, C.
EPI1988977	Belgium	2022-Jan-23	EPI_ISL_10576444	A/Anser_anser/Belgium/1809_0002/2022	Sciensano - Animal Infectious Diseases	Sciensano, Department of Animal Infectious Diseases	Van Borm, Steven; Roupie, Virginie; Lambrecht, Benedicte; Mathijs, Elisabeth; Steensels, Mieke
EPI1930158	Belgium	2021-Nov-04	EPI_ISL_6761101	A/Branta_leucopsis/Belgium/14735_0001/2021	Sciensano - Animal Infectious Diseases	Sciensano, Department of Animal Infectious Diseases	Van Borm, Steven; Roupie, Virginie; Lambrecht, Benedicte; Mathijs, Elisabeth; Steensels, Mieke
EPI1966001	Belgium	2021-Dec-27	EPI_ISL_9161618	A/Gallus_gallus/Belgium/17100_0001/2021	Sciensano - Animal Infectious Diseases	Sciensano, Department of Animal Infectious Diseases	Van Borm, Steven; Roupie, Virginie; Lambrecht, Benedicte; Mathijs, Elisabeth; Steensels, Mieke
EPI1963857	United Kingdom	2021-Nov-14	EPI_ISL_9029956	A/chicken/England/057314/2021	Animal and Plant Health Agency (APHA)	Animal and Plant Health Agency (APHA)	NA
EPI1963040	Russian Federation	2021-Dec-13	EPI_ISL_9009317	A/chicken/Rostov-on-Don/159-7V/2021	State Research Center of Virology and Biotechnology (VECTOR)	State Research Center of Virology and Biotechnology (VECTOR)	NA
EPI1963032	Russian Federation	2021-Dec-13	EPI_ISL_9009309	A/chicken/Rostov-on-Don/159-6V/2021	State Research Center of Virology and Biotechnology (VECTOR)	State Research Center of Virology and	NA

Segment ID	Country	Collection date	Isolate-ID	Isolate name	Originating Lab	Submitting Lab	Authors
						Biotechnology (VECTOR)	
EPI1963024	Russian Federation	2021-Dec-13	EPI_ISL_9009304	A/chicken/Rostov-on-Don/159-5V/2021	State Research Center of Virology and Biotechnology (VECTOR)	State Research Center of Virology and Biotechnology (VECTOR)	NA
EPI1963016	Russian Federation	2021-Dec-13	EPI_ISL_9009303	A/chicken/Rostov-on-Don/159-4V/2021	State Research Center of Virology and Biotechnology (VECTOR)	State Research Center of Virology and Biotechnology (VECTOR)	NA
EPI1963008	Russian Federation	2021-Dec-13	EPI_ISL_9009302	A/chicken/Rostov-on-Don/159-3V/2021	State Research Center of Virology and Biotechnology (VECTOR)	State Research Center of Virology and Biotechnology (VECTOR)	NA
EPI1963000	Russian Federation	2021-Dec-13	EPI_ISL_9009297	A/chicken/Rostov-on-Don/159-2V/2021	State Research Center of Virology and Biotechnology (VECTOR)	State Research Center of Virology and Biotechnology (VECTOR)	NA
EPI1962992	Russian Federation	2021-Dec-13	EPI_ISL_9009296	A/chicken/Rostov-on-Don/159-1V/2021	State Research Center of Virology and Biotechnology (VECTOR)	State Research Center of Virology and Biotechnology (VECTOR)	NA
EPI1962984	Russian Federation	2021-Dec-02	EPI_ISL_9009294	A/chicken/Stavropol/146-1V/2021	State Research Center of Virology and Biotechnology (VECTOR)	State Research Center of Virology and Biotechnology (VECTOR)	NA
EPI1962976	Russian Federation	2021-Nov-27	EPI_ISL_9009292	A/chicken/Kursk/132-1V/2021	State Research Center of Virology and Biotechnology (VECTOR)	State Research Center of Virology and Biotechnology (VECTOR)	NA
EPI1962968	Russian Federation	2021-Nov-27	EPI_ISL_9009291	A/chicken/Kursk/132-1V/2021	Center of Hygiene and Epidemiology in Kursk Oblast	State Research Center of Virology and Biotechnology (VECTOR)	NA
EPI1951309	Czech Republic	2021-Nov-25	EPI_ISL_8515480	A/chicken/Czech_Republic/23404/2021	State Veterinary Institute Prague	State Veterinary Institute Prague	Alexander,Nagy;Lenka,Cernikova;Martina,Stara
EPI1946086	Belgium	2021-Dec-06	EPI_ISL_7880696	A/Gallus_gallus/Belgium/15977/2021	Sciensano - Animal Infectious Diseases	Sciensano, Department of	Van Borm, Steven; Roupie, Virginie; Lambrecht, Benedicte; Mathijs, Elisabeth; Steensels, Mieke

Segment ID	Country	Collection date	Isolate-ID	Isolate name	Originating Lab	Submitting Lab	Authors
						Animal Infectious Diseases	
EPI1946078	Belgium	2021-Dec-07	EPI_ISL_7880689	A/Gallus_gallus/Belgium/16070_003/2021	Sciensano - Animal Infectious Diseases	Sciensano, Department of Animal Infectious Diseases	Van Borm, Steven; Roupie, Virginie; Lambrecht, Benedicte; Mathijs, Elisabeth; Steensels, Mieke
EPI1942186	Sweden	2021-Nov-30	EPI_ISL_7452805	A/Chicken/Sweden/SVA211130SZ0427/FB290424-IP-1/M-2021	National Veterinary Institute, SVA	National Veterinary Institute	NA
EPI1937647	Poland	2021-Nov-05	EPI_ISL_6931288	A/chicken/Poland/H1940-N/2021	National Veterinary Research Institut Poland, PIWet-PIB	National Veterinary Research Institut Poland, PIWet-PIB	E. Swieton, K. Smietanka
EPI1937639	Poland	2021-Nov-03	EPI_ISL_6931008	A/domestic_goose/Poland/H1931-T1/2021	National Veterinary Research Institut Poland, PIWet-PIB	National Veterinary Research Institut Poland, PIWet-PIB	E. Swieton, K. Smietanka
EPI1951301	Czech Republic	2021-Nov-18	EPI_ISL_8515479	A/goose/Czech_Republic/22608-2/2021	State Veterinary Institute Prague	State Veterinary Institute Prague	Alexander,Nagy;Lenka,Cernikova;Martina,Stara
EPI1951293	Czech Republic	2021-Nov-18	EPI_ISL_8515478	A/goose/Czech_Republic/22608-1/2021	State Veterinary Institute Prague	State Veterinary Institute Prague	Alexander,Nagy;Lenka,Cernikova;Martina,Stara
EPI1942304	Croatia	2021-Nov-18	EPI_ISL_7570634	A/goose/Croatia/107/2021	Croatian Veterinary Institute, Poultry Centre	Croatian Veterinary Institute	Savić, Vladimir
EPI1965994	Belgium	2021-Nov-21	EPI_ISL_9160206	A/Anser_albifrons/Belgium/15465_0010/2021	Sciensano - Animal Infectious Diseases	Sciensano, Department of Animal Infectious Diseases	Van Borm, Steven; Roupie, Virginie; Lambrecht, Benedicte; Mathijs, Elisabeth; Steensels, Mieke
EPI1963865	United Kingdom	2021-Nov-16	EPI_ISL_9029957	A/turkey/England/057679/2021	Animal and Plant Health Agency (APHA)	Animal and Plant Health Agency (APHA)	NA
EPI1959924	United Kingdom	2021-Nov-10	EPI_ISL_8814195	A/turkey/England/056764/2021	Animal and Plant Health Agency (APHA)	Animal and Plant Health Agency (APHA)	NA
EPI1959914	United Kingdom	2021-Nov-06	EPI_ISL_8814146	A/turkey/England/055251/2021	Animal and Plant Health Agency (APHA)	Animal and Plant Health Agency (APHA)	NA
EPI1949326	Sweden	2021-Dec-12	EPI_ISL_8338002	A/Turkey/Sweden/SVA211212SZ0001/FB301013-IP-2/M-2021	National Veterinary Institute, SVA	National Veterinary Institute	'Siamak, Zohari'
EPI1937663	Poland	2021-Nov-08	EPI_ISL_6935584	A/turkey/Poland/H1944-N/2021	National Veterinary Research Institut Poland, PIWet-PIB	National Veterinary Research Institut Poland, PIWet-PIB	E. Swieton, K. Smietanka

Segment ID	Country	Collection date	Isolate-ID	Isolate name	Originating Lab	Submitting Lab	Authors
EPI1937631	Poland	2021-Nov-03	EPI_ISL_6930564	A/turkey/Poland/H1924-T1/2021	National Veterinary Research Institut Poland, PIWet-PIB	National Veterinary Research Institut Poland, PIWet-PIB	E. Swieton, K. Smietanka
EPI1937623	Poland	2021-Nov-02	EPI_ISL_6930238	A/turkey/Poland/H1913-T1/2021	National Veterinary Research Institut Poland, PIWet-PIB	National Veterinary Research Institut Poland, PIWet-PIB	E. Swieton, K. Smietanka
EPI1937615	Poland	2021-Nov-01	EPI_ISL_6929970	A/turkey/Poland/H1911-N/2021	National Veterinary Research Institut Poland, PIWet-PIB	National Veterinary Research Institut Poland, PIWet-PIB	E. Swieton, K. Smietanka
EPI1937607	Poland	2021-Nov-01	EPI_ISL_6929958	A/turkey/Poland/H1910-T3/2021	National Veterinary Research Institut Poland, PIWet-PIB	National Veterinary Research Institut Poland, PIWet-PIB	E. Swieton, K. Smietanka

*All submitters of data can be contacted directly through the GISAID website (https://www.gisaid.org); NA: not available.
